# Metabolomic profiles in night shift workers: A cross-sectional study on hospital female nurses

**DOI:** 10.3389/fpubh.2023.1082074

**Published:** 2023-02-23

**Authors:** Elisa Borroni, Gianfranco Frigerio, Elisa Polledri, Rosa Mercadante, Cristina Maggioni, Luca Fedrizzi, Angela Cecilia Pesatori, Silvia Fustinoni, Michele Carugno

**Affiliations:** ^1^Department of Clinical Sciences and Community Health, University of Milan, Milan, Italy; ^2^Luxembourg Centre for Systems Biomedicine (LCSB), University of Luxembourg, Esch-sur-Alzette, Luxembourg; ^3^Occupational Health Unit, Fondazione IRCCS Ca' Granda Ospedale Maggiore Policlinico, Milan, Italy

**Keywords:** night shift work, nurses, targeted metabolomics, Tobit regression, machine-learning, Random Forest, occupational health

## Abstract

**Background and aim:**

Shift work, especially including night shifts, has been found associated with several diseases, including obesity, diabetes, cancers, and cardiovascular, mental, gastrointestinal and sleep disorders. Metabolomics (an omics-based methodology) may shed light on early biological alterations underlying these associations. We thus aimed to evaluate the effect of night shift work (NSW) on serum metabolites in a sample of hospital female nurses.

**Methods:**

We recruited 46 nurses currently working in NSW in Milan (Italy), matched to 51 colleagues not employed in night shifts. Participants filled in a questionnaire on demographics, lifestyle habits, personal and family health history and work, and donated a blood sample. The metabolome was evaluated through a validated targeted approach measuring 188 metabolites. Only metabolites with at least 50% observations above the detection limit were considered, after standardization and log-transformation. Associations between each metabolite and NSW were assessed applying Tobit regression models and Random Forest, a machine-learning algorithm.

**Results:**

When comparing current vs. never night shifters, we observed lower levels of 21 glycerophospholipids and 6 sphingolipids, and higher levels of serotonin (+171.0%, 95%CI: 49.1–392.7), aspartic acid (+155.8%, 95%CI: 40.8–364.7), and taurine (+182.1%, 95%CI: 67.6–374.9). The latter was higher in former vs. never night shifters too (+208.8%, 95%CI: 69.2–463.3). Tobit regression comparing ever (i.e., current + former) and never night shifters returned similar results. Years worked in night shifts did not seem to affect metabolite levels. The Random-Forest algorithm confirmed taurine and aspartic acid among the most important variables in discriminating current vs. never night shifters.

**Conclusions:**

This study, although based on a small sample size, shows altered levels of some metabolites in night shift workers. If confirmed, our results may shed light on early biological alterations that might be related to adverse health effects of NSW.

## 1. Introduction

Shift work (SW) refers to any organization of work hours that differ from the traditional diurnal work period (from 7:00 a.m. to 6:00 p.m.) (1), including evening, night, and early morning shifts, as well as fixed or rotating schedules (2, 3). In particular, night shift work (NSW) refers to any kind of work that covers at least 3 h of work between 11:00 p.m. and 6:00 a.m. (4, 5). In industrialized countries, SW and NSW are common work schedules (6). Indeed, according to the U.S. Bureau of Labor Statistics, ~16% of employees surveyed in 2017–2018 followed SW schedules, including 6% of evening shifts workers and 4% of night shifts workers (7).

SW, especially if including night shifts, has been found to be associated with several diseases, e.g., cardiovascular diseases (8), cancers (9), metabolic disorders such as obesity (10, 11) and type 2 diabetes (12, 13), sleep disturbances (14), gastrointestinal disorders (15), and impaired mental health (16). However, the underlying mechanisms are not fully understood. Some might be mediated by psychosocial stress deriving from interference with social rhythms, but there are indications also suggesting that disruption of normal sleep-wake cycle (circadian rhythm) following SW leads to neuroendocrine and cardiometabolic stress, curtailed and disturbed sleep, and, as a consequence, altered immune functioning and cellular stress (17, 18).

In recent years, omics-based approaches have shown great potential to shed light on mechanisms underlying diseases and their possible association with exposure to relevant risk factors through the identification of biomarkers. Metabolomics, one of the omics-based methodologies, refers to the techniques used to quantify the metabolites present within a cell, tissue or organism (19). These techniques are mainly divided into two strategies: i.e., untargeted and targeted metabolomics. Targeted metabolomics is the measurement of defined groups of chemically characterized and biochemically annotated metabolites (20).

To our knowledge, only a few studies investigated the effects of shift work on the human metabolome. One measured plasma metabolites in 49 male workers at the beginning and end of a rotating shift schedule including nights and observed an association between NSW and alterations in several metabolites (21). Two were laboratory studies aimed at evaluating the impact of simulated 3- (22) and 4-day (23) night shift schedules on the metabolic profile of healthy volunteers, with a particular focus on sleep/wake and feeding/fasting cycles. Other two studies compared shifters and non-shifters. The first one analyzed urinary metabolites, and found altered long-chain acylcarnitines, three amino acids, and one sphingomyelin (24) in night shift workers as compared to day workers, based on both crude and adjusted models. The second study evaluated serum metabolites and identified 76 of them in shift workers (including L- tryptophan, acylcarnitines, and several fatty acids) which may represent important biomarkers of impaired lipid metabolism, leading to weight gain and central obesity (25). As such, evidence on this topic is still limited and further studies are needed. The aim of the present study is thus to evaluate the effect of NSW on serum metabolites in a sample of female nurses, using a targeted metabolomics approach.

## 2. Materials and methods

### 2.1. Study population, personal data, and biological samples

Procedures for recruitment of the study population and collection of personal data and biological samples have been described elsewhere (26). Briefly, 46 female nurses working in night shifts at the Fondazione IRCCS Ca' Granda Ospedale Maggiore Policlinico in Milan, Italy, were recruited on a voluntary basis and matched by age and length of services to 51 colleagues not working in night shifts. Inclusion criteria were Caucasian ethnicity, age 30–45 years and length of service ≥1 year. After signing informed consent, all participants filled in a questionnaire on demographics, lifestyle habits, personal and family health history, and work history (with a particular focus on SW) and donated a 12 mL blood sample. The sample was drawn in the morning, at the end of the night shift for night shifters (7:15–7:45 a.m.) and at the beginning of the working day for day shifters (8:30–9:00 a.m.), to try to maximize potential differences between the two groups. The metabolomics profile (see below) could not be assessed for six subjects, and we thus performed our analyses on a total of 91 nurses.

The study was conducted according to the guidelines of the Declaration of Helsinki and approved by the Institutional Review Board of the Policlinico Hospital (approval number 702_2015).

### 2.2. Metabolomic analysis

The metabolomic profile was assessed with a validated targeted metabolomics approach, implementing liquid chromatography coupled to tandem mass spectrometry (LC-MS/MS), and using the AbsoluteIDQ p180 kit (Biocrates Life Sciences AG AbsoluteIDQ^®^ p180 Kit, Innsbruck, Austria), which benefits of an established good interlaboratory reproducibility (27). Briefly, the serum samples were placed on a 96-well plate pre-loaded with the isotopic labeled internal standards, along with a phosphate buffer solution as blank sample, a calibration curve (7 levels), and three levels of quality control samples. Two different plates were implemented for this study. The sample preparation consisted in the derivatization of amino acids and biogenic amines with phenyl isothiocyanate, evaporation, extraction with 5 mM ammonium acetate in methanol, centrifugation, and dilution. Amino acids and biogenic amines were separated and analyzed through an analytical column before the mass spectrometry (LC-MS/MS), while lipids and the hexose were analyzed with a simple flow injection analysis (FIA-MS/MS). A total of 188 metabolites were measured, including 21 amino acids, 21 biogenic amines, the sum of hexoses, 40 acylcarnitine, 15 sphingolipids (SM), and 90 glycerophospholipids among which 14 lysophosphatidylcholines (LysoPC), 38 diacylphosphatidylcholine (PC aa), and 38 acylalkylphosphatidylcholine (PC ae). Further instrumental and analytical details have been previously reported (28).

### 2.3. Statistical analysis

Differences in the distribution of the main adjustment variables across categories of NSW were assessed through the analysis of variance (ANOVA) for age and BMI (continuous) and chi-squared test for smoking habit (categorical).

Metabolomic data (from both LC-MS/MS and FIA-MS/MS) were batch-normalized through the MetIDQ software (Biocrates) using, for each metabolite, the median values of three repetitions of a quality control (reference sample) analyzed on the same plate, according to the manufacturer's instructions (29). Only metabolites with at least 50% of the observations above the limit of detection (LOD) were considered for the statistical analyses. Among these, each remaining value below the LOD was replaced with a value equal to the minimum LOD (specific for each metabolite). Metabolite concentrations were then log-transformed (base e) and standardized (each value subtracted by the mean and divided by the standard deviation).

To visualize how metabolites correlate with each other, we performed network analyses where metabolites were considered as nodes, and correlation coefficients obtained from each pair of metabolites as edges; the Fruchterman-Reingold force-directed layout algorithm was used, and the values of r were set as edge weights; only statistically significant correlations with *r* > 0.4 were considered and metabolites with no connection were not considered.

To assess the association between each metabolite and NSW, we applied Tobit censored linear regression models, which are useful to estimate linear relationships when considering dependent variables with left- or right-censoring (30, 31): in the present work, we considered metabolite concentrations lower than LOD as left-censored. We built a Tobit model for each metabolite, with the metabolite concentration as dependent variable, and NSW as the main independent variable. NSW was modeled both as current or former vs. never NSW and as ever (i.e., current + former) vs. never NSW. As a sensitivity analysis, we stratified current night shifters according to their shift schedule (see below). We also considered “number of years worked in night shifts” as a variable of interest (equal to 0 in never night shifters). Adjustment variables considered *a priori* as potential confounders were body mass index (BMI) (kg/m^2^), age, plate (plate 1 or 2: i.e., which of the two 96-well plates the serum sample was loaded on during sample preparation for metabolomic analyses), and smoking habit (current vs. former/never smokers). The models assessing the association between metabolites and number of years in night shifts also included the variable “never vs. ever night shift.” Before implementing all models, we imputed the few values missing from our database (one for age, three for BMI, three for smoking habit) using the k-nearest neighbors algorithm (k-NN) (32) with a *k*-value = 9 (32). From each model, we estimated the standardized beta coefficients and calculated the percent variation (Δ%) using the following formula: (exp(β)−1) x 100, where β is the regression coefficient representing the variation in the metabolite level for a unit increase in the independent variable. The *p*-values were adjusted for multiple testing by controlling the false discovery rate (FDR) according to the method of Benjamini and Hochberg (33) and a FDR *p*-value lower than 0.1 was considered statistically significant. To have a visual representation of the Tobit models, Volcano plots were created, assigning a dot to each molecule and plotting the Δ% vs. the negative logarithm of the FDR *p*-value.

A confirmatory analysis was also conducted applying a supervised machine-learning algorithm called Random Forest (RF). A RF consists of many unpruned individual decision trees that operate as an ensemble. Individual trees are grown by bootstrapping a random sample of the original data set and by selecting at random, at each node, a small group of input variables to split on. Results from different decision trees are, subsequently, averaged to make final predictions (34). We used RF to classify subjects into current or former vs. never night shifters and into ever (i.e., current + former) vs. never night shifters, considering correlations among metabolites.

K-fold cross-validation, a statistical method for evaluating a machine-learning model and testing its performance, was applied to assess RF performances and to tune parameters in order to obtain optimal predictions. In the present study, based on 5-fold cross-validation results, we implemented RF algorithms setting the number of trees at 10,000, and the number of variables from which to choose at each node at 11 (i.e., the approximate square root of the total number of metabolites included in the analysis).

Variables importance scores were then calculated. They are RF-derived measures that facilitate results interpretation by ranking the importance of each feature (i.e., metabolite), and can be computed mainly through two methods: (1) Mean Decrease Accuracy, indicating how much the accuracy (i.e., the number of data points out of all data points which are correctly predicted) decreases when the interested variable is excluded; and (2) Mean Decrease Gini, indicating how much the Gini score (which calculates the probability of a specific feature to be classified incorrectly when selected at random) decreases when a variable is chosen to split a node. The larger the scores, the greater the importance of a variable (34). We evaluated variable importance, using both above-cited methods, in order to produce more accurate results.

All statistical analyses were performed using R (R version 4.1.2, R Foundation, Vienna, Austria) (35) with the Rstudio interface (Version 1.4.1717, RStudio Inc., Boston, MA, USA) and the packages “tidyverse” (36), “VIM,” “AER” (37), “tidygraph,” “ggraph” (38, 39), and “randomForest” (40).

## 3. Results

Mean age of our study subjects was similar across categories of night shift work, ranging from 35.1 years in current shift workers to 36.8 years in former shift workers. The majority of current night shifters (67%) followed a counterclockwise, very rapidly rotating schedule (A), in details: day 1: morning (6:00 a.m.−2:00 p.m. or 7:00 a.m.−2:00 p.m.); day 2: either morning or afternoon (2:00 p.m.−10:00 p.m. or 2:00 p.m.−9:00 PM); day 3: both morning and night (10:00 p.m.−6:00 a.m. or 9:00 p.m.−7:00 a.m.), followed by three rest days (72 h). Eight nurses followed a clockwise, rapidly rotating schedule (B), in details: day 1: morning (7:00 a.m.−2:00 p.m.); day 2: afternoon (2:00 p.m.−9:00 p.m.); day 3: night (9:00 p.m.−7:00 a.m.), followed by two or three rest days (48–72 h). Only five nurses worked on a 12 h schedule (C): day – day – night – (night) – rest – rest. For one subject the information was not available. Never night shift workers had a lower BMI compared to former and current shift workers (*p* = 0.057). Percent of current smokers increased from 19% among never night shifters to about 33% in current night shifters (Table 1). Descriptive statistics of metabolite concentrations are reported in Supplementary Table S1.

**Table 1:** Characteristics of the study population stratified by categories of night shift work.

**Characteristic**	**Never night shift workers**	**Former night shift workers**	**Current night shift workers**	*P* ^*^
*N*	26	22	43	
**Type of night shift work** ^**^				
Schedule A—*N* (%)	-	-	29 (67)	
Schedule B—*N* (%)	-	-	8 (19)	
Schedule C—*N* (%)	-	-	5 (12)	
Missing—*N* (%)	-	-	1 (2)	
**Age**				
Mean ± SD	36.6 ± 5.4	36.8 ± 5.4	35.1 ± 5.4	0.382
Missing	0	0	1	
**BMI**				
Mean ± SD	21.4 ± 2.4	23.2 ± 3.9	23.1 ± 3.0	0.057
Missing	0	0	2	
**Smoking habit**				
Former/never smokers—*N* (%)	21 (81)	14 (64)	27 (63)	
Current smokers—*N* (%)	5 (19)	7 (32)	14 (33)	0.389
Missing—*N* (%)	0 (-)	1 (4)	2 (4)	

SD, standard deviation; ^*^from ANOVA (age, BMI) and chi-squared test (smoking habit); ^**^schedule A, counterclockwise, very rapidly rotating schedule; schedule B, clockwise, rapidly rotating schedule; schedule C, 12 h schedule.

Network analysis (Supplementary Figure S1) mainly showed that (i) metabolites belonging to the same category are highly correlated, (ii) serotonin is correlated with taurine, and (iii) taurine is also correlated with aspartic acid.

**Figure 1:**
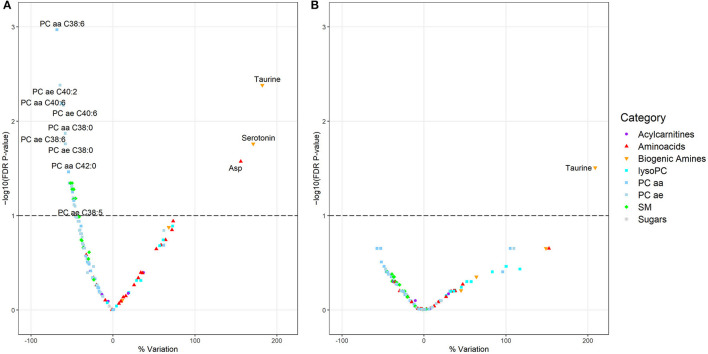
**(A, B)** Volcano plots showing the results of the Tobit linear regression models considering the metabolites (dependent variables) in relation to night shift work: current vs. never night shift workers **(A)** and former vs. never night shift workers **(B)**. The models are adjusted for BMI, age, plate, and smoking habit. Each dot represents a metabolite and is displayed based on the percentage variation of its concentration (x-axis) vs. the negative logarithm (base 10) of the FDR *p*-value (y-axis). The dashed line represents a FDR *p*-value equal to 0.1.

When comparing current vs. never night shift workers (Figure 1A), 6 SM and several glycerophospholipids, among which 12 PC aa and 9 PC ae, were significantly decreased; while taurine, serotonin, and aspartic acids were significantly increased (with a percent variation of +182.1, +171.0, and +155.8%, respectively). When comparing former vs. never night shift workers (Figure 1B), only taurine emerged as a significantly different metabolite, with a percent variation of +208.8%. The Tobit regression comparing ever (i.e., current + former) vs. never night shift workers returned similar results (Supplementary Figure S2). Comparable findings were also observed when stratifying current night shifters by shift schedule and focusing on nurses following schedule A (Supplementary Figure S3A). Only four metabolites were found to be significantly altered in night shift workers following schedule B (Supplementary Figure S3B) while no alteration was observed when inspecting shift schedule C (Supplementary Figure S3C). No metabolite was found to be associated with increasing number of years worked in night shifts (Supplementary Figure S4). Complete results from Tobit regression models are reported in Supplementary Table S2.

**Figure 2:**
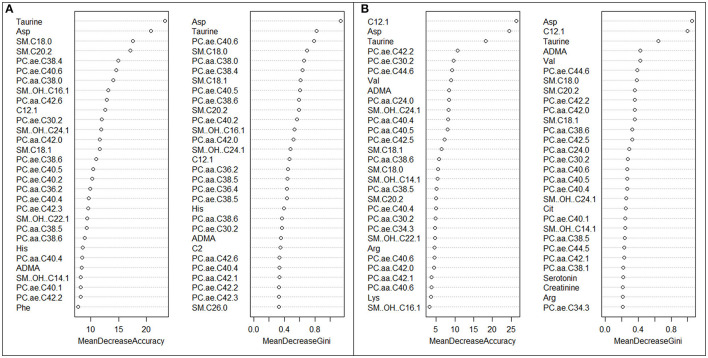
**(A, B)** Variable importance scores plots of the 30 most important metabolites in predicting current vs. never night shift workers **(A)** and former vs. never night shift workers **(B)**, according to both Mean Decrease Accuracy and Mean Decrease Gini.

Figure 2A shows variable importance scores from the RF algorithm for current vs. never night shift workers. Taurine and aspartic acid were the most important variables discriminating subjects in the two groups, according to both Mean Decrease Accuracy and Mean Decrease Gini. Several PC aa and some PC ae were found to be among the 30 most important metabolites. Figure 2B reports variable importance scores for former vs. never night shift workers: C12.1, aspartic acid and taurine were found to be the three most important metabolites, according to both indexes. ADMA, and several PC aa, PC ae, and sphingolipids were also observed among the 30 most important metabolites. Again, when pooling current and former night shifters, we obtained similar results (Supplementary Figure S5).

In Supplementary Table S3, 5-fold cross-validation results are reported. RF performance was high in the analysis of current vs. never night shift workers as both sensitivity and specificity were above 0.80, while it was medium-low for former vs. never shifters. When analyzing ever vs. never night shifters, sensitivity was 0.80 while specificity was 0.65.

## 4. Discussion

In the present study, we evaluated the association between exposure to night shift work and metabolites levels, using a targeted metabolomic approach, in a sample of 91 nurses. In particular, when compared to never night shifters, current night shift workers had higher levels of taurine, serotonin and aspartic acid, while lower levels of several glycerophospholipids and sphingolipids. Similar results were observed in ever night shift workers, while the observed associations disappeared when comparing former shift workers to never night shifters, except for taurine levels. Findings across type of night shift schedule, although mostly confirming the overall results, were hampered by the very small number of subjects following schedules B and C. Number of years worked in night shifts did not impact the levels of metabolites.

Serum metabolites in shift workers were previously investigated on 60 subjects from China (25). Shift workers had altered levels of several lipids, including some glycerophospholipids and sphingolipids, as observed in our study. However, authors also found altered levels of some amines and androgens, and they did not observe any change in serotonin and taurine levels. Differences might relate to the fact that, in the Chinese study, shifters worked two shifts not including the night (i.e., 7:00 a.m.−3:00 p.m.; 3:00 p.m.−11:00 p.m.): as such, their occupational exposure cannot be directly compared to the one in our study. In addition, the applied statistical techniques were different: Huang and colleagues used linear regression models in combination with an Orthogonal Projections to Latent Structures Discriminant Analysis (OPLS-DA), while we used Tobit linear regression models in combination with Random Forests.

A second study (21) investigating male workers, which rotated through 3 weeks of night shifts (10:00 p.m.−6:00 a.m.), followed by 3 weeks of evening shifts (2:00 p.m.−10:00 p.m.) and 3 weeks of early morning shifts (6:00 a.m.−2:00 p.m.), did observe alterations in some lipids (e.g., glycerophospholipids and lysophospholipids) associated with night shifts (i.e., somehow similarly to our results). Nonetheless, the shift scheme is hardly comparable to that experienced by our study population which, in addition, consists of female workers only.

Two other studies conducted in experimental settings simulated night shifts protocols on a small number of healthy volunteers and collected blood samples at repeated time points (22, 23). Both concluded that the observed rhythmicity of several metabolites was driven mainly by behaviors imposed by the simulated shift schedule rather than by the central circadian clock. Notwithstanding the peculiar differences characterizing the experimental settings of these investigations, their findings do confirm the relevant role of night shift work in influencing health, in particular for what concerns metabolic imbalance.

We observed higher levels of taurine in both current and former night shifters when compared to never night shifters. To exclude this finding could be related to energy drink use, we inspected the distribution of the available variable “drinks other than coffee” and found no differences across categories of night shift (chi-squared *p*-value = 0.78). Previous investigations documented higher levels of taurine in both humans and rats during periods of sleep deprivation (41–43), a condition typically related to night shift work (44). In fact, it seems that increased levels of taurine activate the extrasynaptic GABA (A) receptors in the mouse ventrobasal thalamus (45), an area involved in the regulation of the transitions between sleep and wakefulness (46).

Current night shifters showed also higher levels of serotonin. This neurotransmitter is another important factor involved in sleep/wake regulation, functioning primarily to promote wakefulness (42). In addition, altered levels of both serotonin and taurine have been found to be involved in depression onset (47–49). This is particularly interesting in light of a recent meta-analysis, which estimated a 33% increased risk of depressive symptoms associated with shift work, that rose to more than 70% when restricting the analyses to female workers (16). In our study, we found serotonin to be positively associated with night shift work in Tobit regression models only and not in the Random Forests analysis: this inconsistency may be explained by the high correlation existing between serotonin and taurine.

Current night shift workers had lower levels of several glycerophospholipids. Decreased levels of such lipids were also found in the plasma sample of breast cancer patients (50), reflecting a higher activity of phospholipase A2 (PLA2), an important pro-inflammatory mediator (51). On the other hand, higher concentrations of several glycerophospholipids were associated with decreased risk of prostate cancer subtypes, especially those in advanced stage (52). Positive associations between night shift work and both these cancer types have been mentioned by IARC in supporting the evaluation of NSW as probably carcinogenic to humans (group 2A carcinogen) (5). Higher levels of phospholipids were also documented by several investigations to be negatively associated with metabolic diseases, as altered concentrations of such lipids were found in subjects with dyslipidemia, hypertension, obesity, insulin resistance or type 2 diabetes (53–58). In particular, it was found that elevated levels of phosphatidylcholines showed a possibly anti-inflammatory role under different conditions (e.g., oxidative stress and ulcerative colitis) (59–61). Indeed, phosphatidylcholines inhibit the upregulation of the inflammatory cytokines tumor necrosis factor alpha and interleukin-6 as well as the actin-assembly in phagosomes and macrophages (59, 61). In this multifaceted scenario, night shift work emerges as a potentially relevant player in the development of metabolic disorders and cancers.

Another class of lipids we found to be decreased in night shift workers are the sphingolipids. Lower levels of sphingolipids were found in patients with a diagnosis of major depressive disorder: Demirkan and colleagues identified significant negative associations between the sphingomyelin (SM) ratio 23:1 to SM 16:0 and a psychometric depression measure (Center for Epidemiological Studies-Depression Scale: CES-D) (62). However, subsequent analysis of an independent replication dataset did not confirm previous results. Another study from Liu and colleagues found that several differential lipid species were significantly correlated with depression severity measured by the Hamilton Depression Scale (HAMD) (63). Moreover, rats exposed to chronic stress had reduced sphingomyelin and dihydrosphingomyelin levels in the prefrontal cortex (PFC) (64). This region vulnerability fits with previous studies showing that PFC is the brain region displaying major lipid alterations after the use of maprotiline, an antidepressant. In our study population, only four subjects (ever shifters) declared a prolonged use of psychotropic drugs (not better specified). Decreased levels of sphingolipids were also found in subjects with dyslipidemia (56) and with diabetes mellitus (53), even if some other publications showed opposite results (54), indicating that there is no clear pattern between sphingolipids and metabolic disorders.

Levels of aspartic acid were found to be elevated in current shift workers. This is a relative new result. Indeed, few publications on elevated levels of aspartate were published. A study from Guevara-Cruz conducted in Mexico found that levels of aspartate were elevated in a 20-years-old population affected by obesity and insulin resistance (65). Similar results were also found in a study conducted by Yamada and colleagues, in a Japanese non-diabetic population (66). Moreover, higher levels of aspartic acid were also found in subjects with epilepsy, as compared to disease-free controls (67). However, the available research is still too limited and further studies are needed to draw robust conclusions.

The present study has some strengths. This is one of the few studies investigating the effects of night shift work on human serum metabolome. Blood samples were collected within a relatively narrow time window (7:15–9:00 a.m.) to minimize the 24-h variations of metabolites levels (68). Moreover, we evaluated the investigated associations using two different statistical methodologies (i.e., Tobit linear regression models and Random Forests) to make more robust conclusions. The first ones allow to adjust for individual confounders but are not able to consider correlations among the different metabolites. On the contrary, Random Forests are able to take into proper consideration inter-metabolite correlations but not to adjust for individual confounders. As such, the use of both methods provides a more comprehensive picture of our findings. In addition, we considered observations with non-determinable metabolite levels as left-censored, and applied Tobit linear regression models which are particularly adequate when dealing with dependent variables with censored values (30).

This study has also some limitations. First, it is a cross-sectional study, thus preventing to assess causality. Second, sample size is relatively small, not allowing to obtain optimal prediction results in the application of machine-learning algorithms and to thoroughly investigate the potential role of night shift schedule in influencing our findings. Third, our sample was entirely composed of women, preventing the possibility to detect sex-related differences which have been previously observed with metabolomics data, even if in experimental sleep-deprivation settings (42, 69). Fourth, all variables including the exposure of interest (i.e., shift work status) as well as all the confounders were self-reported, although the absence of a pathologic outcome should allow to avoid major distortions. Last, given the limited set of available information, we were not able to fully disentangle whether the observed metabolic alterations were related directly to NSW (i.e., by modification of the endogenous circadian clock) or rather due to changes in behavioral (e.g., sleep/wake or feeding/fasting) cycles.

In conclusion, our study, although based on a small sample size, shows an alteration of metabolites levels in night shift workers when compared to never night shifters. In particular, serum concentrations of taurine, serotonin, and aspartic acid were higher, while those of several glycerophospholipids and sphingolipids were lower, independently from number of years worked in night shifts. These findings may shed light on early biological alterations that might be related to adverse health effects of NSW, such as metabolic disorders, cancers, and mental diseases. However, further studies including a larger sample size and male workers are needed to confirm our results.

## Data availability statement

The raw data supporting the conclusions of this article will be made available by the authors, without undue reservation.

## Ethics statement

The studies involving human participants were reviewed and approved by Institutional Review Board of the Policlinico Hospital (approval number 702_2015). The patients/participants provided their written informed consent to participate in this study.

## Author contributions

Conceptualization: MC, GF, CM, ACP, and SF. Methodology: EB, GF, and MC. Software and formal analysis: EB, GF, and LF. Validation: LF and MC. Investigation and writing—original draft preparation: EB and GF. Resources and data curation: GF, EP, and RM. Writing—review and editing: MC, EP, RM, CM, LF, ACP, and SF. Visualization: MC and GF. Supervision: ACP, SF, and MC. Project administration: CM. Funding acquisition: MC and CM. All authors have read and agreed to the published version of the manuscript.
