# Supplementary material for: Metabolomic profiles in night shift workers: A cross-sectional study on hospital female nurses
Source: Front Public Health. 2023 Feb 23;11:1082074. doi: 10.3389/fpubh.2023.1082074 (PMC9999616; doi:10.3389/fpubh.2023.1082074)

**Figure S1**: Network analysis performed considering metabolites as nodes and correlation coefficients (r) obtained from each pair of metabolites as edges. The Fruchterman–Reingold force-directed layout algorithm was used, and the edge weights were set on the value of r. Only statistically significant correlations with r > 0.4 were considered and metabolites with no connections were removed.


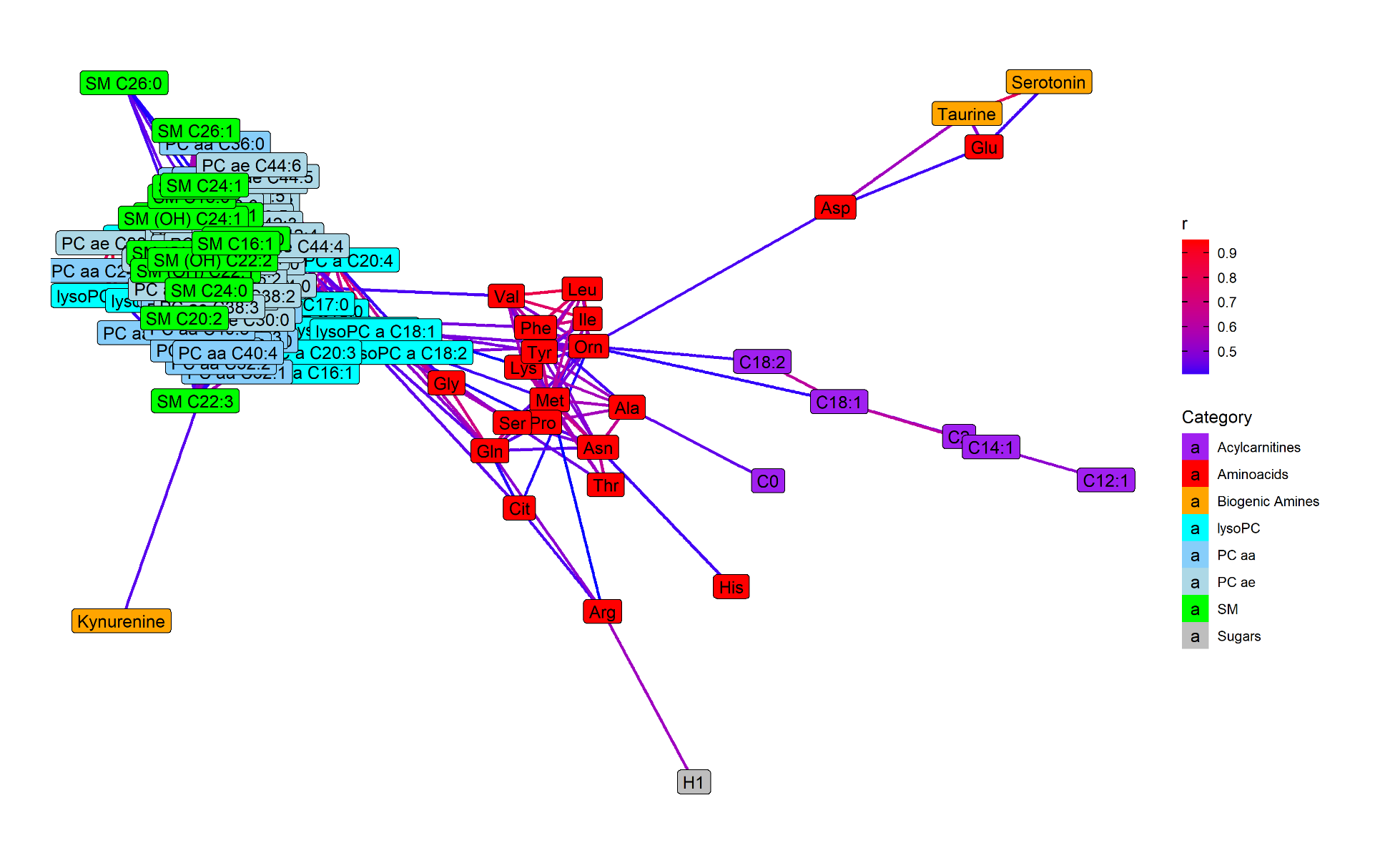


**Figure S2:** Volcano plots showing the results of the Tobit linear regression models considering the metabolites (dependent variables) in relation to night shift work: ever (i.e., current + former) vs. never night shift workers. The models are adjusted for BMI, age, plate, and smoking habit. Each dot represents a metabolite and is displayed based on the percentage variation of its concentration (x-axis) vs. the negative logarithm (base 10) of the FDR *p*-value (y-axis). The dashed line represents a FDR *p*-value equal to 0.1.


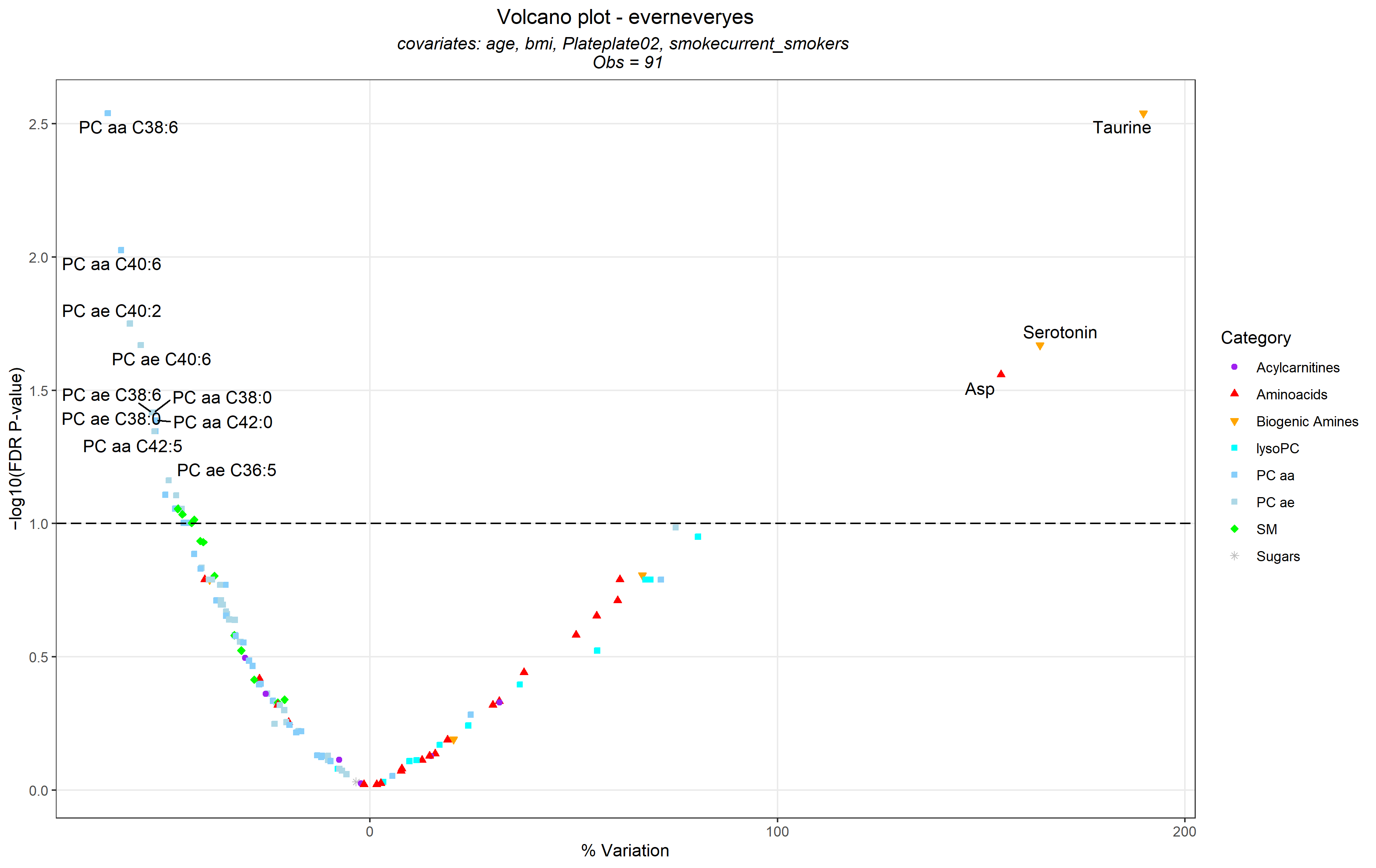


**Figure S3A-C:** Volcano plots showing the results of the Tobit linear regression models comparing metabolites (dependent variables) among never night shifters vs. current night shift workers, stratified by shift schedule: schedule A (top panel), schedule B (middle panel), and schedule C (bottom panel). The models are adjusted for BMI, age, plate, and smoking habit. Each dot represents a metabolite and is displayed based on the percentage variation of its concentration (x-axis) vs. the negative logarithm (base 10) of the FDR *p*-value (y-axis). The dashed line represents a FDR *p*-value equal to 0.1.

| 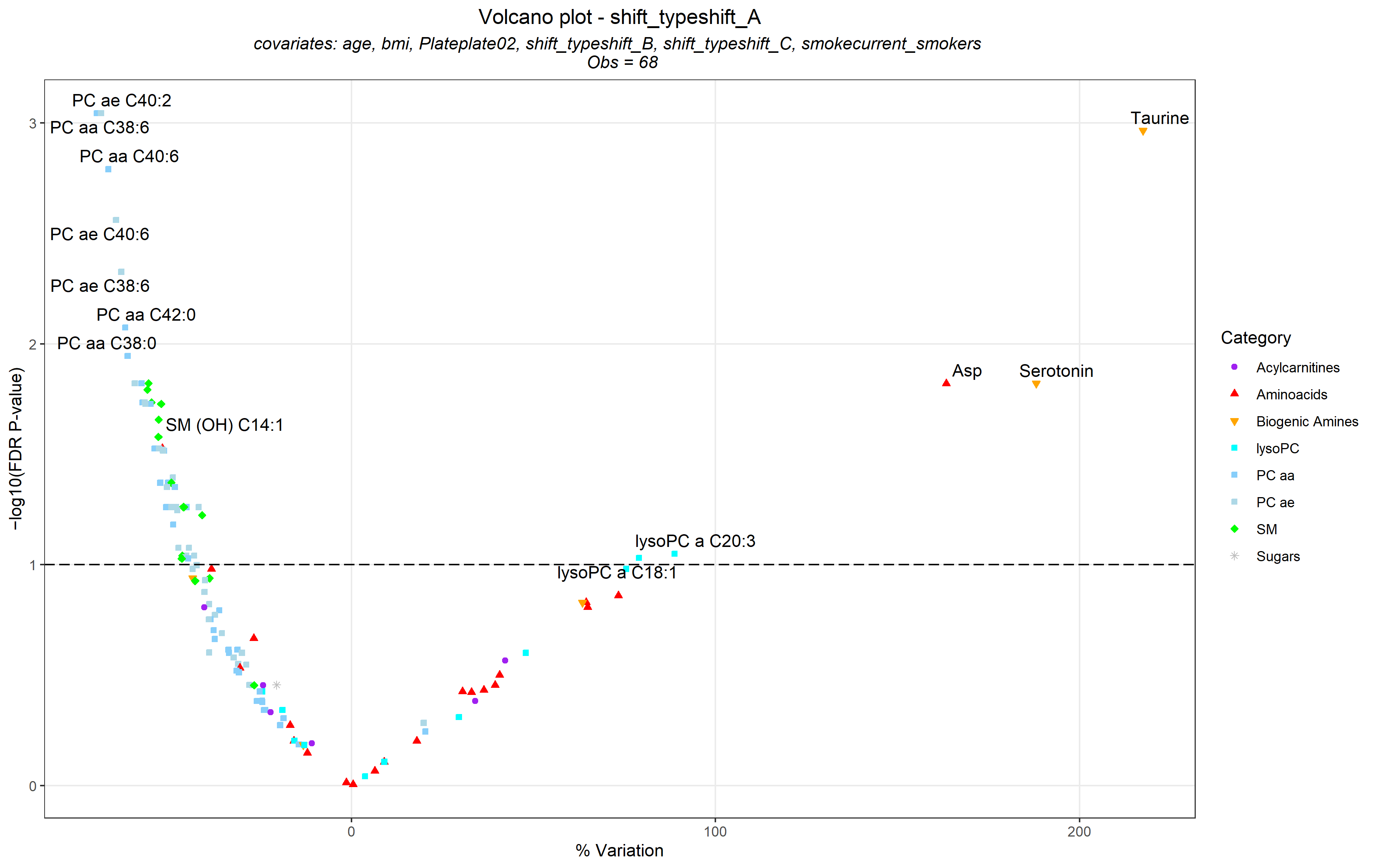 |
| --- |
| 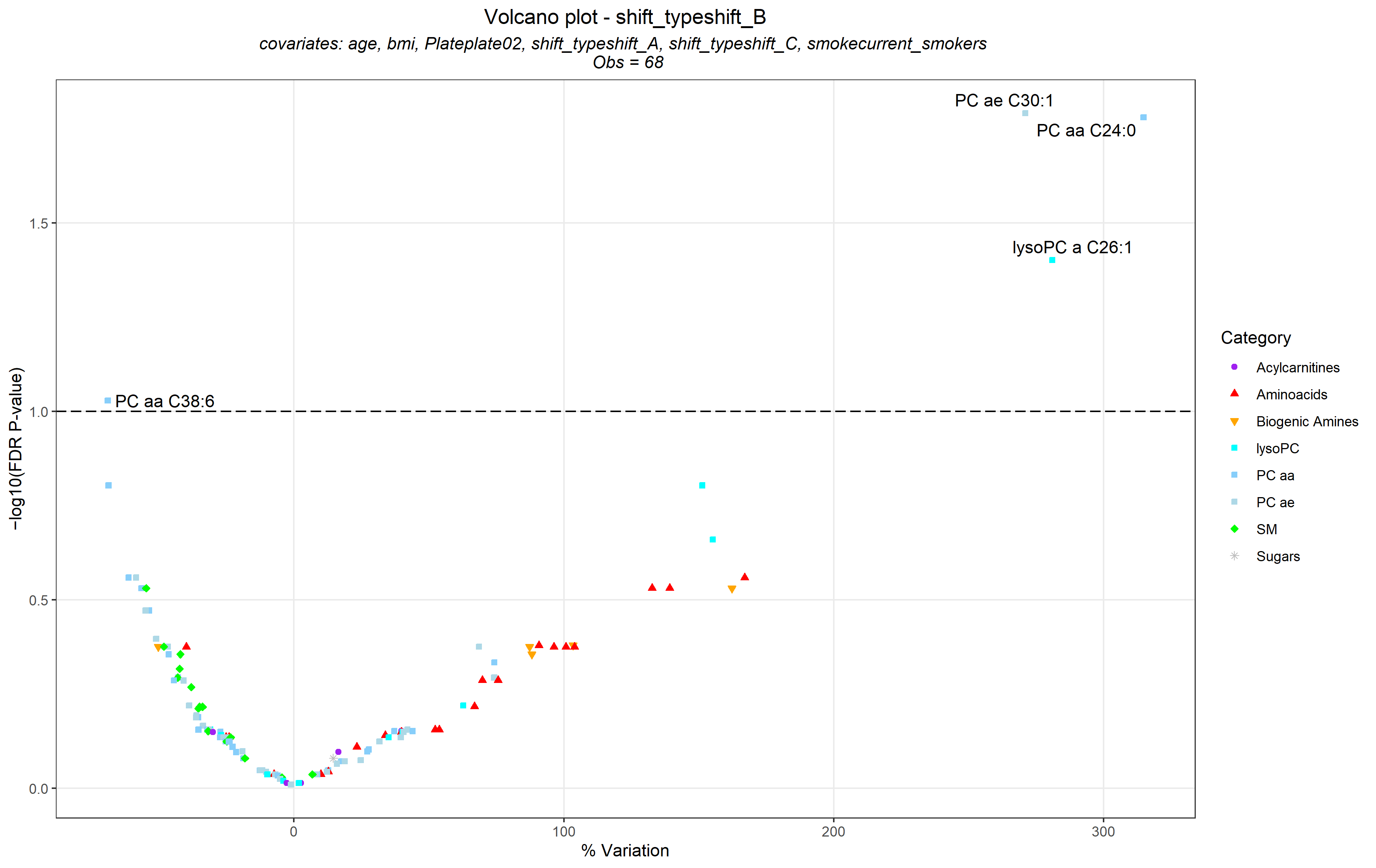 |
| 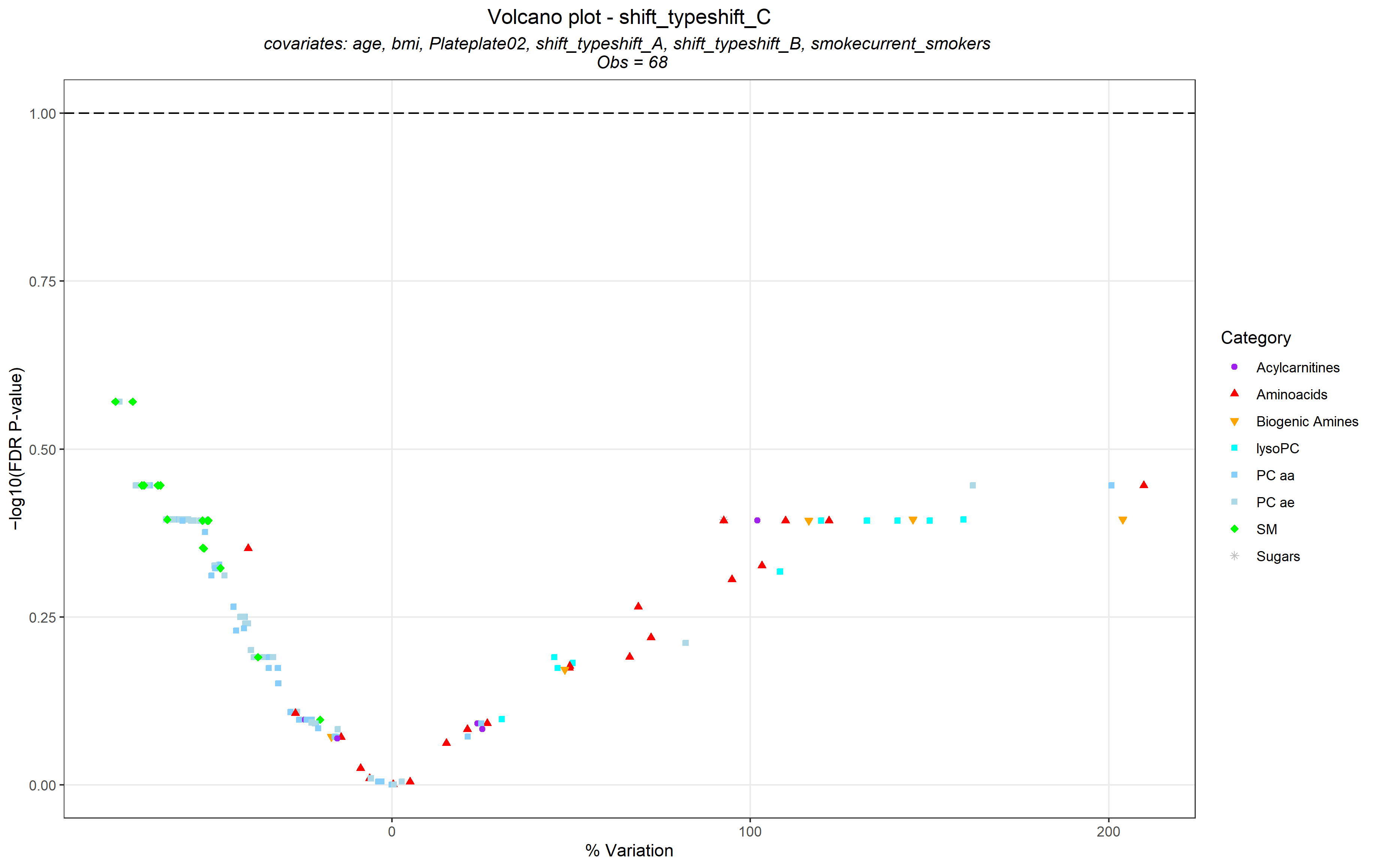 |

**C**

**B**

**A**

**Figure S4:** Volcano plots showing the results of the Tobit linear regression models considering the metabolites (dependent variables) in relation to the number of years worked in night shifts. The models are adjusted for BMI, age, type of shift work (ever vs. never), plate, and smoking habit. Each dot represents a metabolite and is displayed based on the percentage variation of its concentration (x-axis) vs. the negative logarithm (base 10) of the FDR *p*-value (y-axis). The dashed line represents a FDR *p*-value equal to 0.1.

**
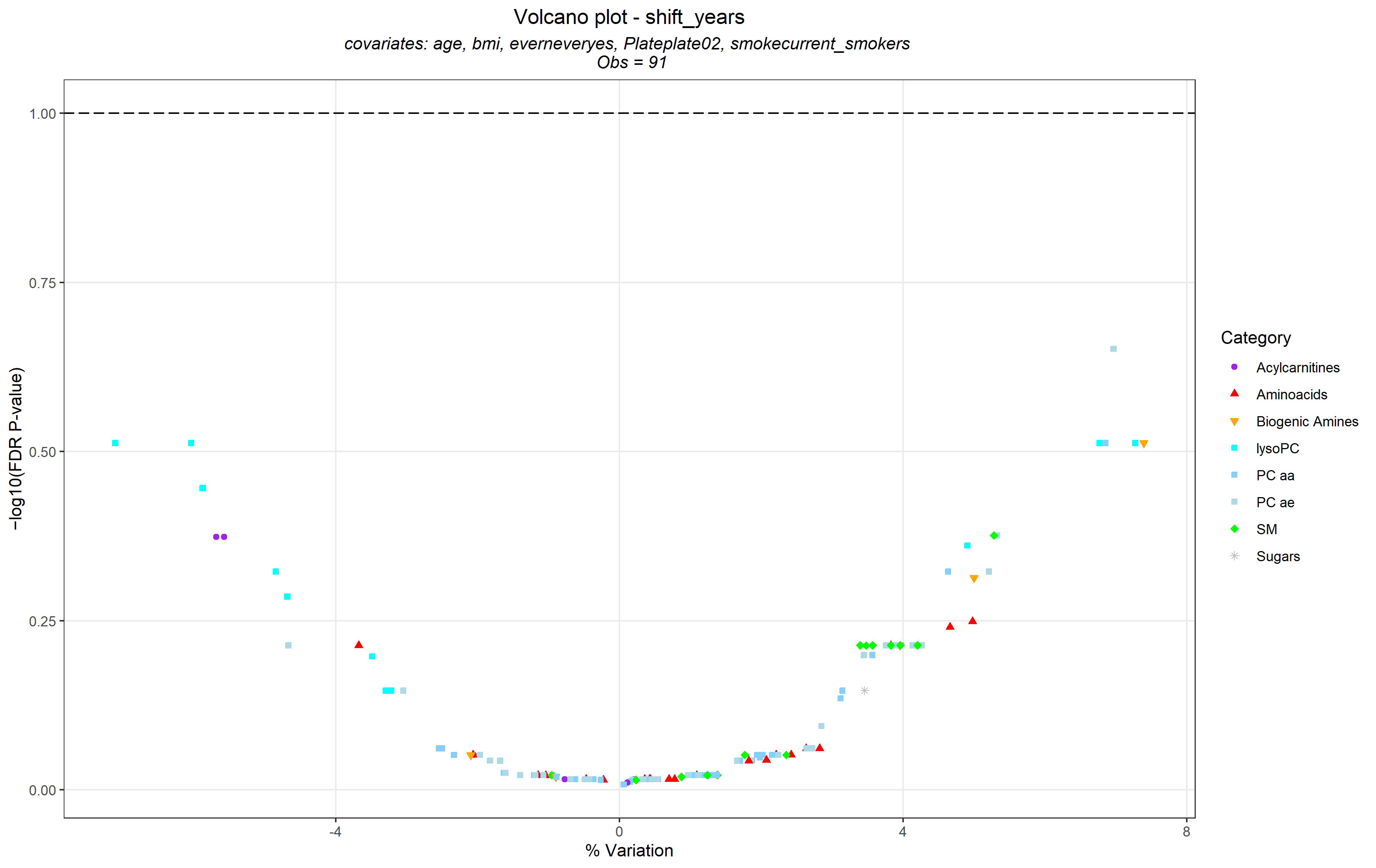
**

**Figure S5**: Variable Importance Scores Plots of the 30 most important metabolites in predicting ever vs. never night shift workers, according to both Mean Decrease Accuracy and Mean Decrease Gini


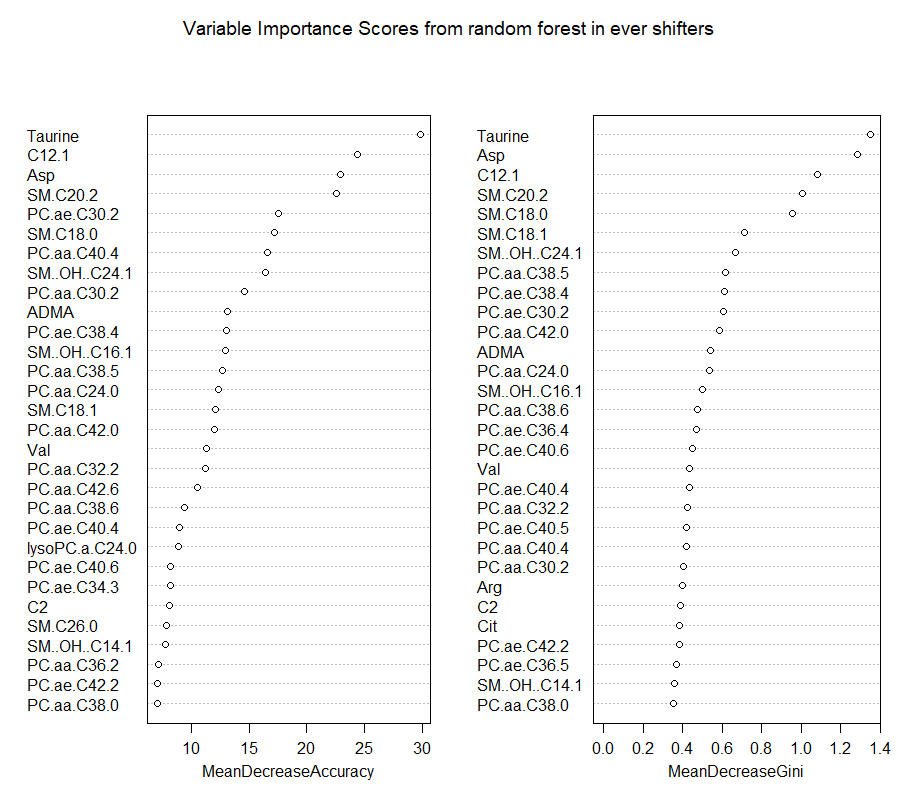

Supplement: Supplementary file 1 [file Data_Sheet_1.ZIP › Borroni_NSW_Metabolome_SupplMaterial/Borroni_NSW_Metabolome_SupplFigures_v1.docx]
