# Supplementary material for: Metabolomic profiles in night shift workers: A cross-sectional study on hospital female nurses
Source: Front Public Health. 2023 Feb 23;11:1082074. doi: 10.3389/fpubh.2023.1082074 (PMC9999616; doi:10.3389/fpubh.2023.1082074)
Supplement: Supplementary file 1 [file Data_Sheet_1.ZIP › Borroni_NSW_Metabolome_SupplMaterial/Borroni_NSW_Metabolome_Table S1.docx]

**Table S1**: Descriptive statistics of metabolite concentrations measured in the study subjects’ sera, expressed in µM, and stratified by categories of night shift work. For each molecule, median, 5^th^ and 95^th^ percentile are reported, along with the percentage of samples greater than the limit of detection (LOD).

| **Metabolite^*^** | **Never night shift workers (N = 26)** | **Former night shift workers (N = 22)** | **Current night shift workers (N = 43)** |
| --- | --- | --- | --- |
| **C0** |  |  |  |
| Median | 30.4 | 32.1 | 28.8 |
| 5th-95th percentile | 23.2 - 37.3 | 21.2 - 42.6 | 21.5 - 38.5 |
| %>LOD | 100 | 100 | 100 |
| **C2** |  |  |  |
| Median | 4.82 | 4.46 | 3.71 |
| 5th-95th percentile | 2.61 - 8.17 | 3.07 - 6.49 | 2.64 - 7.37 |
| %>LOD | 100 | 100 | 100 |
| **C3** |  |  |  |
| Median | 0.4 | 0.4 | 0.4 |
| 5th-95th percentile | 0.4 - 0.476 | 0.4 - 0.4 | 0.4 - 0.4 |
| %>LOD | 8 | 0 | 5 |
| **C3-DC (C4-OH)** |  |  |  |
| Median | 0.21 | 0.21 | 0.21 |
| 5th-95th percentile | 0.21 - 0.21 | 0.21 - 0.21 | 0.21 - 0.21 |
| %>LOD | 0 | 0 | 0 |
| **C3-OH** |  |  |  |
| Median | 0.241 | 0.241 | 0.241 |
| 5th-95th percentile | 0.241 - 0.241 | 0.241 - 0.241 | 0.241 - 0.241 |
| %>LOD | 0 | 0 | 0 |
| **C3:1** |  |  |  |
| Median | 0.073 | 0.073 | 0.073 |
| 5th-95th percentile | 0.073 - 0.073 | 0.073 - 0.073 | 0.073 - 0.073 |
| %>LOD | 0 | 0 | 0 |
| **C4** |  |  |  |
| Median | 0.4 | 0.4 | 0.4 |
| 5th-95th percentile | 0.4 - 0.4 | 0.4 - 0.472 | 0.4 - 0.4 |
| %>LOD | 0 | 9 | 5 |
| **C4:1** |  |  |  |
| Median | 0.104 | 0.104 | 0.104 |
| 5th-95th percentile | 0.104 - 0.104 | 0.104 - 0.104 | 0.104 - 0.104 |
| %>LOD | 0 | 0 | 0 |
| **C5** |  |  |  |
| Median | 0.4 | 0.4 | 0.4 |
| 5th-95th percentile | 0.4 - 0.4 | 0.4 - 0.4 | 0.4 - 0.4 |
| %>LOD | 0 | 0 | 0 |
| **C5-DC (C6-OH)** |  |  |  |
| Median | 0.124 | 0.124 | 0.124 |
| 5th-95th percentile | 0.124 - 0.124 | 0.124 - 0.124 | 0.124 - 0.124 |
| %>LOD | 0 | 0 | 0 |
| **C5-M-DC** |  |  |  |
| Median | 0.123 | 0.123 | 0.123 |
| 5th-95th percentile | 0.123 - 0.123 | 0.123 - 0.123 | 0.123 - 0.123 |
| %>LOD | 0 | 0 | 0 |
| **C5-OH (C3-DC-M)** |  |  |  |
| Median | 0.203 | 0.203 | 0.203 |
| 5th-95th percentile | 0.203 - 0.203 | 0.203 - 0.203 | 0.203 - 0.203 |
| %>LOD | 0 | 0 | 0 |
|  |  |  |  |
| **Metabolite^*^** | **Never**  **night shift workers** | **Former**  **night shift workers** | **Current**  **night shift workers** |
| **C5:1** |  |  |  |
| Median | 0.111 | 0.111 | 0.111 |
| 5th-95th percentile | 0.111 - 0.111 | 0.111 - 0.111 | 0.111 - 0.111 |
| %>LOD | 0 | 0 | 0 |
| **C5:1.DC** |  |  |  |
| Median | 0.15 | 0.15 | 0.15 |
| 5th-95th percentile | 0.15 - 0.15 | 0.15 - 0.15 | 0.15 - 0.15 |
| %>LOD | 0 | 0 | 0 |
| **C6 (C4:1-DC)** |  |  |  |
| Median | 0.095 | 0.095 | 0.095 |
| 5th-95th percentile | 0.095 - 0.095 | 0.095 - 0.095 | 0.095 - 0.095 |
| %>LOD | 0 | 0 | 0 |
| **C6:1** |  |  |  |
| Median | 0.08 | 0.08 | 0.08 |
| 5th-95th percentile | 0.08 - 0.08 | 0.08 - 0.08 | 0.08 - 0.08 |
| %>LOD | 0 | 0 | 0 |
| **C7-DC** |  |  |  |
| Median | 0.111 | 0.111 | 0.111 |
| 5th-95th percentile | 0.111 - 0.111 | 0.111 - 0.111 | 0.111 - 0.111 |
| %>LOD | 0 | 0 | 0 |
| **C8** |  |  |  |
| Median | 0.2 | 0.2 | 0.2 |
| 5th-95th percentile | 0.2 - 0.346 | 0.2 - 0.2 | 0.2 - 0.2 |
| %>LOD | 8 | 0 | 5 |
| **C9** |  |  |  |
| Median | 0.15 | 0.15 | 0.15 |
| 5th-95th percentile | 0.15 - 0.15 | 0.15 - 0.15 | 0.15 - 0.15 |
| %>LOD | 0 | 0 | 0 |
| **C10** |  |  |  |
| Median | 0.3 | 0.3 | 0.3 |
| 5th-95th percentile | 0.3 - 0.587 | 0.3 - 0.455 | 0.3 - 0.527 |
| %>LOD | 31 | 32 | 28 |
| **C10:1** |  |  |  |
| Median | 0.14 | 0.14 | 0.14 |
| 5th-95th percentile | 0.14 - 0.18 | 0.14 - 0.14 | 0.14 - 0.153 |
| %>LOD | 15 | 5 | 7 |
| **C10:2** |  |  |  |
| Median | 0.229 | 0.229 | 0.229 |
| 5th-95th percentile | 0.229 - 0.229 | 0.229 - 0.229 | 0.229 - 0.229 |
| %>LOD | 0 | 0 | 0 |
| **C12** |  |  |  |
| Median | 0.4 | 0.4 | 0.4 |
| 5th-95th percentile | 0.4 - 0.4 | 0.4 - 0.4 | 0.4 - 0.4 |
| %>LOD | 0 | 0 | 0 |
| **C12-DC** |  |  |  |
| Median | 1.23 | 1.23 | 1.23 |
| 5th-95th percentile | 1.23 - 1.23 | 1.23 - 1.23 | 1.23 - 1.23 |
| %>LOD | 0 | 0 | 0 |
| **C12:1** |  |  |  |
| Median | 0.104 | 0.258 | 0.256 |
| 5th-95th percentile | 0.104 - 0.373 | 0.229 - 0.324 | 0.104 - 0.35 |
| %>LOD | 46 | 100 | 91 |
|  |  |  |  |
| **Metabolite^*^** | **Never**  **night shift workers** | **Former**  **night shift workers** | **Current**  **night shift workers** |
| **C14** |  |  |  |
| Median | 0.4 | 0.4 | 0.4 |
| 5th-95th percentile | 0.4 - 0.4 | 0.4 - 0.4 | 0.4 - 0.4 |
| %>LOD | 0 | 0 | 0 |
| **C14:1** |  |  |  |
| Median | 0.062 | 0.074 | 0.074 |
| 5th-95th percentile | 0.038 - 0.192 | 0.038 - 0.121 | 0.038 - 0.199 |
| %>LOD | 81 | 59 | 65 |
| **C14:1-OH** |  |  |  |
| Median | 0.047 | 0.047 | 0.047 |
| 5th-95th percentile | 0.047 - 0.047 | 0.047 - 0.047 | 0.047 - 0.047 |
| %>LOD | 0 | 0 | 0 |
| **C14:2** |  |  |  |
| Median | 0.039 | 0.039 | 0.039 |
| 5th-95th percentile | 0.039 - 0.047 | 0.039 - 0.039 | 0.039 - 0.039 |
| %>LOD | 8 | 0 | 5 |
| **C14:2-OH** |  |  |  |
| Median | 0.032 | 0.032 | 0.032 |
| 5th-95th percentile | 0.032 - 0.032 | 0.032 - 0.032 | 0.032 - 0.032 |
| %>LOD | 0 | 0 | 0 |
| **C16** |  |  |  |
| Median | 0.4 | 0.4 | 0.4 |
| 5th-95th percentile | 0.4 - 0.4 | 0.4 - 0.4 | 0.4 - 0.4 |
| %>LOD | 0 | 0 | 0 |
| **C16-OH** |  |  |  |
| Median | 0.055 | 0.055 | 0.055 |
| 5th-95th percentile | 0.055 - 0.055 | 0.055 - 0.055 | 0.055 - 0.055 |
| %>LOD | 0 | 0 | 0 |
| **C16:1** |  |  |  |
| Median | 0.046 | 0.046 | 0.046 |
| 5th-95th percentile | 0.046 - 0.046 | 0.046 - 0.046 | 0.046 - 0.046 |
| %>LOD | 4 | 5 | 2 |
| **C16:1-OH** |  |  |  |
| Median | 0.044 | 0.044 | 0.044 |
| 5th-95th percentile | 0.044 - 0.044 | 0.044 - 0.044 | 0.044 - 0.044 |
| %>LOD | 0 | 0 | 0 |
| **C16:2** |  |  |  |
| Median | 0.044 | 0.044 | 0.044 |
| 5th-95th percentile | 0.044 - 0.044 | 0.044 - 0.044 | 0.044 - 0.044 |
| %>LOD | 0 | 0 | 0 |
| **C16:2-OH** |  |  |  |
| Median | 0.042 | 0.042 | 0.042 |
| 5th-95th percentile | 0.042 - 0.042 | 0.042 - 0.042 | 0.042 - 0.042 |
| %>LOD | 0 | 0 | 0 |
| **C18** |  |  |  |
| Median | 0.4 | 0.4 | 0.4 |
| 5th-95th percentile | 0.4 - 0.4 | 0.4 - 0.4 | 0.4 - 0.4 |
| %>LOD | 0 | 0 | 0 |
| **C18:1** |  |  |  |
| Median | 0.106 | 0.112 | 0.122 |
| 5th-95th percentile | 0.057 - 0.142 | 0.074 - 0.15 | 0.066 - 0.176 |
| %>LOD | 92 | 95 | 95 |
|  |  |  |  |
| **Metabolite^*^** | **Never**  **night shift workers** | **Former**  **night shift workers** | **Current**  **night shift workers** |
| **C18:1-OH** |  |  |  |
| Median | 0.087 | 0.087 | 0.087 |
| 5th-95th percentile | 0.087 - 0.087 | 0.087 - 0.087 | 0.087 - 0.087 |
| %>LOD | 0 | 0 | 0 |
| **C18:2** |  |  |  |
| Median | 0.044 | 0.044 | 0.056 |
| 5th-95th percentile | 0.044 - 0.064 | 0.044 - 0.085 | 0.044 - 0.073 |
| %>LOD | 38 | 45 | 60 |
| **Ala** |  |  |  |
| Median | 410 | 418 | 354 |
| 5th-95th percentile | 272 - 558 | 215 - 563 | 226 - 537 |
| %>LOD | 100 | 100 | 100 |
| **Arg** |  |  |  |
| Median | 72 | 52 | 59 |
| 5th-95th percentile | 39 - 122 | 32 - 96 | 27 - 96 |
| %>LOD | 100 | 100 | 100 |
| **Asn** |  |  |  |
| Median | 55.8 | 53.9 | 55.4 |
| 5th-95th percentile | 37.5 - 76.5 | 37.9 - 72.3 | 40.4 - 74.1 |
| %>LOD | 100 | 100 | 100 |
| **Asp** |  |  |  |
| Median | 6 | 8 | 7.2 |
| 5th-95th percentile | 5 - 8.5 | 5 - 9.9 | 5.6 - 10.5 |
| %>LOD | 58 | 86 | 95 |
| **Cit** |  |  |  |
| Median | 27.2 | 25.1 | 22.2 |
| 5th-95th percentile | 14.7 - 39.6 | 17.7 - 35.7 | 14.9 - 35.6 |
| %>LOD | 100 | 100 | 100 |
| **Gln** |  |  |  |
| Median | 650 | 650 | 689 |
| 5th-95th percentile | 512 - 914 | 537 - 826 | 512 - 844 |
| %>LOD | 100 | 100 | 100 |
| **Glu** |  |  |  |
| Median | 34 | 31 | 34 |
| 5th-95th percentile | 19 - 62 | 18 - 47 | 20 - 62 |
| %>LOD | 100 | 100 | 100 |
| **Gly** |  |  |  |
| Median | 264 | 262 | 256 |
| 5th-95th percentile | 143 - 356 | 145 - 454 | 139 - 444 |
| %>LOD | 100 | 100 | 100 |
| **His** |  |  |  |
| Median | 112 | 104 | 102 |
| 5th-95th percentile | 79 - 136 | 85 - 144 | 79 - 121 |
| %>LOD | 100 | 100 | 100 |
| **Ile** |  |  |  |
| Median | 73 | 84 | 99 |
| 5th-95th percentile | 57 - 119 | 62 - 114 | 65 - 128 |
| %>LOD | 100 | 100 | 100 |
| **Leu** |  |  |  |
| Median | 154 | 174 | 184 |
| 5th-95th percentile | 114 - 224 | 125 - 244 | 133 - 247 |
| %>LOD | 100 | 100 | 100 |
|  |  |  |  |
| **Metabolite^*^** | **Never**  **night shift workers** | **Former**  **night shift workers** | **Current**  **night shift workers** |
| **Lys** |  |  |  |
| Median | 276 | 262 | 259 |
| 5th-95th percentile | 188 - 324 | 216 - 341 | 193 - 323 |
| %>LOD | 100 | 100 | 100 |
| **Met** |  |  |  |
| Median | 27.4 | 26.3 | 29.1 |
| 5th-95th percentile | 19 - 45.6 | 21.2 - 37.1 | 20.1 - 40.8 |
| %>LOD | 100 | 100 | 100 |
| **Orn** |  |  |  |
| Median | 88 | 99 | 100 |
| 5th-95th percentile | 54 - 134 | 62 - 135 | 61 - 161 |
| %>LOD | 100 | 100 | 100 |
| **Phe** |  |  |  |
| Median | 78 | 79 | 82 |
| 5th-95th percentile | 60 - 104 | 64 - 102 | 69 - 102 |
| %>LOD | 100 | 100 | 100 |
| **Pro** |  |  |  |
| Median | 234 | 232 | 233 |
| 5th-95th percentile | 144 - 369 | 146 - 391 | 135 - 369 |
| %>LOD | 100 | 100 | 100 |
| **Ser** |  |  |  |
| Median | 135 | 140 | 147 |
| 5th-95th percentile | 102 - 223 | 98 - 207 | 100 - 203 |
| %>LOD | 100 | 100 | 100 |
| **Thr** |  |  |  |
| Median | 146 | 160 | 158 |
| 5th-95th percentile | 92 - 230 | 100 - 224 | 103 - 222 |
| %>LOD | 100 | 100 | 100 |
| **Trp** |  |  |  |
| Median | 84 | 77 | 84 |
| 5th-95th percentile | 59 - 104 | 65 - 95 | 60 - 104 |
| %>LOD | 100 | 100 | 100 |
| **Tyr** |  |  |  |
| Median | 78.6 | 79.3 | 80.9 |
| 5th-95th percentile | 40.6 - 107.8 | 58.7 - 122.2 | 51.4 - 119.3 |
| %>LOD | 100 | 100 | 100 |
| **Val** |  |  |  |
| Median | 272 | 318 | 295 |
| 5th-95th percentile | 210 - 372 | 194 - 382 | 212 - 373 |
| %>LOD | 100 | 100 | 100 |
| **Ac-Orn** |  |  |  |
| Median | 1 | 1 | 1 |
| 5th-95th percentile | 0.98 - 1.37 | 1 - 3.022 | 1 - 1.632 |
| %>LOD | 27 | 23 | 14 |
| **ADMA** |  |  |  |
| Median | 0.394 | 0.463 | 0.463 |
| 5th-95th percentile | 0.242 - 0.567 | 0.378 - 0.706 | 0.313 - 0.699 |
| %>LOD | 85 | 100 | 98 |
| **Alpha-AAA** |  |  |  |
| Median | 1 | 1 | 1 |
| 5th-95th percentile | 1 - 1 | 1 - 1 | 1 - 1.295 |
| %>LOD | 4 | 5 | 12 |
|  |  |  |  |
| **Metabolite^*^** | **Never**  **night shift workers** | **Former**  **night shift workers** | **Current**  **night shift workers** |
| **c4-OH-Pro** |  |  |  |
| Median | 10 | 10 | 10 |
| 5th-95th percentile | 10 - 10 | 10 - 10 | 10 - 10 |
| %>LOD | 0 | 0 | 0 |
| **Carnosine** |  |  |  |
| Median | 0.5 | 0.5 | 0.5 |
| 5th-95th percentile | 0.5 - 0.5 | 0.5 - 0.5 | 0.5 - 0.5 |
| %>LOD | 0 | 0 | 0 |
| **Creatinine** |  |  |  |
| Median | 64.1 | 58.5 | 59.2 |
| 5th-95th percentile | 49.1 - 78.8 | 50.3 - 69.2 | 45.3 - 72.1 |
| %>LOD | 100 | 100 | 100 |
| **DOPA** |  |  |  |
| Median | 0.5 | 0.5 | 0.5 |
| 5th-95th percentile | 0.5 - 0.5 | 0.5 - 0.5 | 0.5 - 0.5 |
| %>LOD | 0 | 0 | 0 |
| **Dopamine** |  |  |  |
| Median | 1 | 1 | 1 |
| 5th-95th percentile | 1 - 1 | 1 - 1 | 1 - 1 |
| %>LOD | 0 | 0 | 0 |
| **Histamine** |  |  |  |
| Median | 1 | 1 | 1 |
| 5th-95th percentile | 1 - 1 | 1 - 1 | 1 - 1 |
| %>LOD | 0 | 0 | 0 |
| **Kynurenine** |  |  |  |
| Median | 2.83 | 2.77 | 2.7 |
| 5th-95th percentile | 1.73 - 4.05 | 1.86 - 4.04 | 1.91 - 3.71 |
| %>LOD | 100 | 100 | 98 |
| **Met-SO** |  |  |  |
| Median | 10 | 10 | 10 |
| 5th-95th percentile | 10 - 10 | 10 - 10 | 10 - 10 |
| %>LOD | 0 | 0 | 0 |
| **Nitro-Tyr** |  |  |  |
| Median | 1 | 1 | 1 |
| 5th-95th percentile | 1 - 1 | 1 - 1 | 1 - 1 |
| %>LOD | 0 | 0 | 0 |
| **PEA** |  |  |  |
| Median | 0.1 | 0.1 | 0.1 |
| 5th-95th percentile | 0.1 - 0.1 | 0.1 - 0.1 | 0.1 - 0.1 |
| %>LOD | 0 | 0 | 0 |
| **Putrescine** |  |  |  |
| Median | 0.1 | 0.1 | 0.1 |
| 5th-95th percentile | 0.1 - 0.129 | 0.1 - 0.151 | 0.1 - 0.162 |
| %>LOD | 19 | 32 | 35 |
| **SDMA** |  |  |  |
| Median | 2 | 2 | 2 |
| 5th-95th percentile | 2 - 2 | 2 - 2 | 2 - 2 |
| %>LOD | 0 | 0 | 0 |
| **Serotonin** |  |  |  |
| Median | 0.215 | 0.266 | 0.252 |
| 5th-95th percentile | 0.1 - 0.507 | 0.102 - 0.511 | 0.1 - 0.997 |
| %>LOD | 88 | 91 | 88 |
|  |  |  |  |
| **Metabolite^*^** | **Never**  **night shift workers** | **Former**  **night shift workers** | **Current**  **night shift workers** |
| **Spermidine** |  |  |  |
| Median | 0.25 | 0.25 | 0.25 |
| 5th-95th percentile | 0.25 - 0.25 | 0.25 - 0.359 | 0.25 - 0.37 |
| %>LOD | 4 | 9 | 7 |
| **Spermine** |  |  |  |
| Median | 0.25 | 0.25 | 0.25 |
| 5th-95th percentile | 0.25 - 0.252 | 0.234 - 0.335 | 0.244 - 0.361 |
| %>LOD | 12 | 55 | 40 |
| **t4-OH-Pro** |  |  |  |
| Median | 10 | 10 | 10 |
| 5th-95th percentile | 10 - 27.2 | 10 - 18.4 | 10 - 18.4 |
| %>LOD | 27 | 18 | 26 |
| **Taurine** |  |  |  |
| Median | 62.3 | 76.3 | 74.4 |
| 5th-95th percentile | 43.7 - 93.2 | 53.8 - 117.9 | 52.6 - 109.4 |
| %>LOD | 100 | 100 | 100 |
| **Total DMA** |  |  |  |
| Median | 1.25 | 1.25 | 1.25 |
| 5th-95th percentile | 1.25 - 1.25 | 1.25 - 1.25 | 1.25 - 1.25 |
| %>LOD | 0 | 0 | 0 |
| **lysoPC a C14:0** |  |  |  |
| Median | 12.4 | 12.4 | 12.4 |
| 5th-95th percentile | 12.4 - 12.4 | 12.4 - 12.4 | 12.4 - 12.4 |
| %>LOD | 0 | 0 | 0 |
| **lysoPC a C16:0** |  |  |  |
| Median | 84 | 78 | 73 |
| 5th-95th percentile | 53 - 113 | 56 - 109 | 48 - 108 |
| %>LOD | 100 | 100 | 100 |
| **lysoPC a C16:1** |  |  |  |
| Median | 2.43 | 2.34 | 2.09 |
| 5th-95th percentile | 1.26 - 4.13 | 1.62 - 2.98 | 1.31 - 4.34 |
| %>LOD | 100 | 100 | 100 |
| **lysoPC a C17:0** |  |  |  |
| Median | 1.48 | 1.48 | 1.29 |
| 5th-95th percentile | 0.94 - 2.18 | 0.86 - 2.28 | 0.71 - 2.26 |
| %>LOD | 100 | 100 | 100 |
| **lysoPC a C18:0** |  |  |  |
| Median | 25.1 | 23.2 | 21.4 |
| 5th-95th percentile | 11.9 - 35.5 | 15.6 - 36.3 | 11.5 - 30.7 |
| %>LOD | 100 | 100 | 100 |
| **lysoPC a C18:1** |  |  |  |
| Median | 17.5 | 17.6 | 18.4 |
| 5th-95th percentile | 10.8 - 29.3 | 13.6 - 35 | 9.1 - 33.3 |
| %>LOD | 100 | 100 | 100 |
| **lysoPC a C18:2** |  |  |  |
| Median | 26.9 | 25.2 | 29.3 |
| 5th-95th percentile | 13.1 - 43.2 | 16.6 - 45.9 | 11.9 - 51.6 |
| %>LOD | 100 | 100 | 100 |
| **lysoPC a C20:3** |  |  |  |
| Median | 1.99 | 1.92 | 1.95 |
| 5th-95th percentile | 0.98 - 3.24 | 1.32 - 2.78 | 1.03 - 3.65 |
| %>LOD | 100 | 100 | 100 |
|  |  |  |  |
| **Metabolite^*^** | **Never**  **night shift workers** | **Former**  **night shift workers** | **Current**  **night shift workers** |
| **lysoPC a C20:4** |  |  |  |
| Median | 5.35 | 5.41 | 5.08 |
| 5th-95th percentile | 3.46 - 8.18 | 3.07 - 9.19 | 3.15 - 8.64 |
| %>LOD | 100 | 100 | 100 |
| **lysoPC a C24:0** |  |  |  |
| Median | 0.288 | 0.244 | 0.222 |
| 5th-95th percentile | 0.168 - 0.472 | 0.168 - 0.329 | 0.168 - 0.333 |
| %>LOD | 81 | 86 | 72 |
| **lysoPC a C26:0** |  |  |  |
| Median | 0.204 | 0.204 | 0.204 |
| 5th-95th percentile | 0.136 - 0.272 | 0.204 - 0.682 | 0.201 - 0.849 |
| %>LOD | 50 | 9 | 16 |
| **lysoPC a C26:1** |  |  |  |
| Median | 0.118 | 0.121 | 0.091 |
| 5th-95th percentile | 0.062 - 0.28 | 0.065 - 0.24 | 0.055 - 0.2 |
| %>LOD | 100 | 100 | 100 |
| **lysoPC a C28:0** |  |  |  |
| Median | 0.245 | 0.245 | 0.245 |
| 5th-95th percentile | 0.155 - 0.299 | 0.245 - 0.638 | 0.215 - 0.598 |
| %>LOD | 54 | 27 | 19 |
| **lysoPC a C28:1** |  |  |  |
| Median | 0.2 | 0.175 | 0.151 |
| 5th-95th percentile | 0.104 - 0.326 | 0.12 - 0.236 | 0.1 - 0.25 |
| %>LOD | 100 | 100 | 100 |
| **PC aa C24:0** |  |  |  |
| Median | 0.192 | 0.136 | 0.13 |
| 5th-95th percentile | 0.066 - 0.395 | 0.066 - 0.216 | 0.066 - 0.259 |
| %>LOD | 77 | 82 | 81 |
| **PC aa C26:0** |  |  |  |
| Median | 0.801 | 1.195 | 0.801 |
| 5th-95th percentile | 0.719 - 1.572 | 0.801 - 1.695 | 0.801 - 1.728 |
| %>LOD | 58 | 59 | 40 |
| **PC aa C28:1** |  |  |  |
| Median | 2.62 | 2.43 | 2.19 |
| 5th-95th percentile | 2.04 - 3.77 | 1.93 - 3.4 | 1.32 - 3.38 |
| %>LOD | 100 | 100 | 100 |
| **PC aa C30:0** |  |  |  |
| Median | 3.79 | 3.61 | 3.12 |
| 5th-95th percentile | 2.24 - 5.93 | 2.36 - 6.23 | 1.84 - 6.64 |
| %>LOD | 100 | 100 | 100 |
| **PC aa C30:2** |  |  |  |
| Median | 0.066 | 0.068 | 0.044 |
| 5th-95th percentile | 0.017 - 0.225 | 0.026 - 0.096 | 0.011 - 0.118 |
| %>LOD | 100 | 100 | 100 |
| **PC aa C32:0** |  |  |  |
| Median | 13.15 | 12.2 | 11.5 |
| 5th-95th percentile | 9.52 - 20.5 | 9.18 - 16.77 | 8.36 - 17.82 |
| %>LOD | 100 | 100 | 100 |
| **PC aa C32:1** |  |  |  |
| Median | 11.6 | 10.3 | 10.1 |
| 5th-95th percentile | 6 - 38.3 | 5 - 18.8 | 4.9 - 24.6 |
| %>LOD | 100 | 100 | 100 |
|  |  |  |  |
| **Metabolite^*^** | **Never**  **night shift workers** | **Former**  **night shift workers** | **Current**  **night shift workers** |
| **PC aa C32:2** |  |  |  |
| Median | 3.79 | 2.93 | 2.93 |
| 5th-95th percentile | 1.03 - 6.89 | 1.81 - 5.08 | 1.44 - 5.07 |
| %>LOD | 100 | 100 | 100 |
| **PC aa C32:3** |  |  |  |
| Median | 0.358 | 0.312 | 0.29 |
| 5th-95th percentile | 0.252 - 0.517 | 0.208 - 0.431 | 0.182 - 0.432 |
| %>LOD | 100 | 100 | 100 |
| **PC aa C34:1** |  |  |  |
| Median | 185 | 161 | 162 |
| 5th-95th percentile | 133 - 275 | 127 - 214 | 113 - 226 |
| %>LOD | 100 | 100 | 100 |
| **PC aa C34:2** |  |  |  |
| Median | 296 | 262 | 264 |
| 5th-95th percentile | 208 - 370 | 236 - 346 | 220 - 323 |
| %>LOD | 100 | 100 | 100 |
| **PC aa C34:3** |  |  |  |
| Median | 12.35 | 9.93 | 10.4 |
| 5th-95th percentile | 6.41 - 22.13 | 6.61 - 14.88 | 6.64 - 16.17 |
| %>LOD | 100 | 100 | 100 |
| **PC aa C34:4** |  |  |  |
| Median | 1.335 | 1.24 | 1.27 |
| 5th-95th percentile | 0.716 - 2.613 | 0.605 - 2.076 | 0.531 - 1.968 |
| %>LOD | 100 | 100 | 100 |
| **PC aa C36:0** |  |  |  |
| Median | 2.46 | 1.77 | 1.55 |
| 5th-95th percentile | 0.81 - 4.25 | 1.03 - 3.23 | 0.88 - 3.01 |
| %>LOD | 100 | 100 | 100 |
| **PC aa C36:1** |  |  |  |
| Median | 56.3 | 47.3 | 44.6 |
| 5th-95th percentile | 29.6 - 85.6 | 30.1 - 75.7 | 31.8 - 68.5 |
| %>LOD | 100 | 100 | 100 |
| **PC aa C36:2** |  |  |  |
| Median | 226 | 204 | 195 |
| 5th-95th percentile | 130 - 305 | 164 - 262 | 165 - 263 |
| %>LOD | 100 | 100 | 100 |
| **PC aa C36:3** |  |  |  |
| Median | 137.5 | 125 | 120 |
| 5th-95th percentile | 72.5 - 211.8 | 95.3 - 199 | 84.1 - 162.6 |
| %>LOD | 100 | 100 | 100 |
| **PC aa C36:4** |  |  |  |
| Median | 170 | 164 | 154 |
| 5th-95th percentile | 131.2 - 279 | 116.3 - 198.8 | 90.8 - 223.4 |
| %>LOD | 100 | 100 | 100 |
| **PC aa C36:5** |  |  |  |
| Median | 18.05 | 14.25 | 12 |
| 5th-95th percentile | 9.61 - 34.65 | 6.98 - 33.09 | 6.29 - 23.84 |
| %>LOD | 100 | 100 | 100 |
| **PC aa C36:6** |  |  |  |
| Median | 0.92 | 0.683 | 0.603 |
| 5th-95th percentile | 0.45 - 1.355 | 0.352 - 1.339 | 0.296 - 1.039 |
| %>LOD | 100 | 100 | 100 |
|  |  |  |  |
| **Metabolite^*^** | **Never**  **night shift workers** | **Former**  **night shift workers** | **Current**  **night shift workers** |
| **PC aa C38:0** |  |  |  |
| Median | 3.12 | 2.59 | 2.15 |
| 5th-95th percentile | 1.65 - 4.51 | 1.71 - 3.67 | 1.33 - 4.42 |
| %>LOD | 100 | 100 | 100 |
| **PC aa C38:1** |  |  |  |
| Median | 0.92 | 0.6 | 0.44 |
| 5th-95th percentile | 0.2 - 1.42 | <LOD - 1.38 | <LOD - 1.32 |
| %>LOD | 96 | 82 | 88 |
| **PC aa C38:3** |  |  |  |
| Median | 49.2 | 41.9 | 39.1 |
| 5th-95th percentile | 24.8 - 70.8 | 26 - 58.5 | 27.5 - 55 |
| %>LOD | 100 | 100 | 100 |
| **PC aa C38:4** |  |  |  |
| Median | 94.2 | 83.5 | 79.4 |
| 5th-95th percentile | 59.8 - 141.5 | 46.3 - 109.6 | 46.2 - 112.8 |
| %>LOD | 100 | 100 | 100 |
| **PC aa C38:5** |  |  |  |
| Median | 48.9 | 38.8 | 36.9 |
| 5th-95th percentile | 27.5 - 59.8 | 22.5 - 50.3 | 21.1 - 49.6 |
| %>LOD | 100 | 100 | 100 |
| **PC aa C38:6** |  |  |  |
| Median | 76.3 | 61.6 | 53.7 |
| 5th-95th percentile | 40.4 - 118.8 | 37.8 - 99.9 | 31.7 - 89.2 |
| %>LOD | 100 | 100 | 100 |
| **PC aa C40:1** |  |  |  |
| Median | 0.53 | 0.53 | 0.53 |
| 5th-95th percentile | 0.53 - 0.57 | 0.53 - 0.53 | 0.53 - 0.53 |
| %>LOD | 19 | 5 | 5 |
| **PC aa C40:2** |  |  |  |
| Median | 0.23 | 0.21 | 0.2 |
| 5th-95th percentile | 0.15 - 0.32 | 0.13 - 0.29 | 0.13 - 0.28 |
| %>LOD | 100 | 100 | 100 |
| **PC aa C40:3** |  |  |  |
| Median | 0.42 | 0.36 | 0.33 |
| 5th-95th percentile | 0.26 - 0.59 | 0.24 - 0.47 | 0.24 - 0.47 |
| %>LOD | 100 | 100 | 100 |
| **PC aa C40:4** |  |  |  |
| Median | 2.69 | 2.42 | 2.45 |
| 5th-95th percentile | 1.76 - 4.52 | 1.54 - 3.18 | 1.48 - 3.57 |
| %>LOD | 100 | 100 | 100 |
| **PC aa C40:5** |  |  |  |
| Median | 7.8 | 6.07 | 6.76 |
| 5th-95th percentile | 4.76 - 11.68 | 3.98 - 8.69 | 4.06 - 9.71 |
| %>LOD | 100 | 100 | 100 |
| **PC aa C40:6** |  |  |  |
| Median | 26.9 | 19.5 | 18.1 |
| 5th-95th percentile | 15.2 - 39.4 | 13.7 - 32.9 | 12 - 33.7 |
| %>LOD | 100 | 100 | 100 |
| **PC aa C42:0** |  |  |  |
| Median | 0.508 | 0.411 | 0.387 |
| 5th-95th percentile | 0.359 - 0.679 | 0.313 - 0.635 | 0.3 - 0.662 |
| %>LOD | 100 | 100 | 100 |
|  |  |  |  |
| **Metabolite^*^** | **Never**  **night shift workers** | **Former**  **night shift workers** | **Current**  **night shift workers** |
| **PC aa C42:1** |  |  |  |
| Median | 0.182 | 0.148 | 0.144 |
| 5th-95th percentile | 0.118 - 0.254 | 0.115 - 0.216 | 0.104 - 0.269 |
| %>LOD | 100 | 100 | 100 |
| **PC aa C42:2** |  |  |  |
| Median | 0.211 | 0.212 | 0.179 |
| 5th-95th percentile | 0.162 - 0.288 | 0.164 - 0.301 | 0.127 - 0.254 |
| %>LOD | 100 | 100 | 100 |
| **PC aa C42:4** |  |  |  |
| Median | 0.148 | 0.131 | 0.134 |
| 5th-95th percentile | 0.094 - 0.202 | 0.106 - 0.176 | 0.094 - 0.172 |
| %>LOD | 100 | 100 | 100 |
| **PC aa C42:5** |  |  |  |
| Median | 0.221 | 0.181 | 0.182 |
| 5th-95th percentile | 0.149 - 0.295 | 0.12 - 0.292 | 0.124 - 0.252 |
| %>LOD | 100 | 100 | 100 |
| **PC aa C42:6** |  |  |  |
| Median | 0.344 | 0.294 | 0.288 |
| 5th-95th percentile | 0.224 - 0.477 | 0.205 - 0.495 | 0.21 - 0.38 |
| %>LOD | 100 | 100 | 100 |
| **PC ae C30:0** |  |  |  |
| Median | 0.342 | 0.331 | 0.295 |
| 5th-95th percentile | 0.256 - 0.503 | 0.213 - 0.498 | 0.177 - 0.483 |
| %>LOD | 100 | 100 | 100 |
| **PC ae C30:1** |  |  |  |
| Median | 0.108 | 0.096 | 0.09 |
| 5th-95th percentile | 0.038 - 0.214 | 0.044 - 0.166 | 0.043 - 0.16 |
| %>LOD | 100 | 100 | 100 |
| **PC ae C30:2** |  |  |  |
| Median | 0.093 | 0.078 | 0.068 |
| 5th-95th percentile | 0.06 - 0.146 | 0.065 - 0.106 | 0.043 - 0.104 |
| %>LOD | 100 | 100 | 100 |
| **PC ae C32:1** |  |  |  |
| Median | 3.02 | 2.63 | 2.44 |
| 5th-95th percentile | 2.2 - 3.84 | 1.75 - 3.58 | 1.76 - 3.53 |
| %>LOD | 100 | 100 | 100 |
| **PC ae C32:2** |  |  |  |
| Median | 0.799 | 0.679 | 0.634 |
| 5th-95th percentile | 0.502 - 1.078 | 0.511 - 0.92 | 0.448 - 0.985 |
| %>LOD | 100 | 100 | 100 |
| **PC ae C34:0** |  |  |  |
| Median | 1.205 | 1.285 | 1.07 |
| 5th-95th percentile | 0.866 - 1.775 | 0.715 - 1.768 | 0.694 - 1.687 |
| %>LOD | 100 | 100 | 100 |
| **PC ae C34:1** |  |  |  |
| Median | 9.59 | 8.57 | 7.7 |
| 5th-95th percentile | 7.07 - 15.9 | 6 - 12.34 | 5.9 - 11.32 |
| %>LOD | 100 | 100 | 100 |
| **PC ae C34:2** |  |  |  |
| Median | 12.15 | 11.3 | 9.25 |
| 5th-95th percentile | 7.66 - 19.32 | 6.1 - 14.08 | 6.79 - 13.91 |
| %>LOD | 100 | 100 | 100 |
|  |  |  |  |
| **Metabolite^*^** | **Never**  **night shift workers** | **Former**  **night shift workers** | **Current**  **night shift workers** |
| **PC ae C34:3** |  |  |  |
| Median | 8.54 | 7.38 | 6.74 |
| 5th-95th percentile | 5.43 - 13.2 | 4.34 - 9.08 | 4.88 - 9.59 |
| %>LOD | 100 | 100 | 100 |
| **PC ae C36:0** |  |  |  |
| Median | 0.742 | 0.645 | 0.614 |
| 5th-95th percentile | 0.519 - 1.09 | 0.478 - 0.944 | 0.462 - 0.815 |
| %>LOD | 100 | 100 | 100 |
| **PC ae C36:1** |  |  |  |
| Median | 6.5 | 5.9 | 5.5 |
| 5th-95th percentile | 4.6 - 10.8 | 4.4 - 7.9 | 3.3 - 8.3 |
| %>LOD | 100 | 100 | 100 |
| **PC ae C36:2** |  |  |  |
| Median | 14.05 | 11.85 | 11.2 |
| 5th-95th percentile | 9.04 - 20.55 | 7.88 - 17.36 | 7.93 - 16.27 |
| %>LOD | 100 | 100 | 100 |
| **PC ae C36:3** |  |  |  |
| Median | 7.44 | 7 | 6.04 |
| 5th-95th percentile | 4.34 - 10.67 | 4.02 - 8.89 | 4.81 - 8.95 |
| %>LOD | 100 | 100 | 100 |
| **PC ae C36:4** |  |  |  |
| Median | 20.9 | 18.3 | 16.6 |
| 5th-95th percentile | 11.8 - 28.57 | 10.56 - 25.71 | 11.93 - 24.28 |
| %>LOD | 100 | 100 | 100 |
| **PC ae C36:5** |  |  |  |
| Median | 12.5 | 10.35 | 9.44 |
| 5th-95th percentile | 7.06 - 16.6 | 6.71 - 13.88 | 6.22 - 14.46 |
| %>LOD | 100 | 100 | 100 |
| **PC ae C38:0** |  |  |  |
| Median | 2.51 | 2.06 | 1.82 |
| 5th-95th percentile | 1.36 - 3.67 | 1.25 - 3.37 | 1.14 - 3.19 |
| %>LOD | 100 | 100 | 100 |
| **PC ae C38:1** |  |  |  |
| Median | 0.35 | 0.36 | 0.3 |
| 5th-95th percentile | 0.05 - 0.72 | 0.05 - 0.59 | 0.05 - 0.67 |
| %>LOD | 88 | 86 | 81 |
| **PC ae C38:2** |  |  |  |
| Median | 1.6 | 1.44 | 1.36 |
| 5th-95th percentile | 1.23 - 2.45 | 0.93 - 2.02 | 0.89 - 1.87 |
| %>LOD | 100 | 100 | 100 |
| **PC ae C38:3** |  |  |  |
| Median | 3.2 | 2.8 | 2.8 |
| 5th-95th percentile | 2.1 - 4.8 | 1.9 - 4.6 | 2 - 3.7 |
| %>LOD | 100 | 100 | 100 |
| **PC ae C38:4** |  |  |  |
| Median | 13.6 | 11.1 | 10.5 |
| 5th-95th percentile | 8.5 - 17.4 | 7.6 - 15 | 7.3 - 14 |
| %>LOD | 100 | 100 | 100 |
| **PC ae C38:5** |  |  |  |
| Median | 17.05 | 15.3 | 13.3 |
| 5th-95th percentile | 9.11 - 21.45 | 10.03 - 19.57 | 9.39 - 18.88 |
| %>LOD | 100 | 100 | 100 |
|  |  |  |  |
| **Metabolite^*^** | **Never**  **night shift workers** | **Former**  **night shift workers** | **Current**  **night shift workers** |
| **PC ae C38:6** |  |  |  |
| Median | 8.39 | 6.67 | 5.77 |
| 5th-95th percentile | 4.24 - 11.37 | 4.08 - 9.62 | 3.78 - 10.24 |
| %>LOD | 100 | 100 | 100 |
| **PC ae C40:1** |  |  |  |
| Median | 1.23 | 1.03 | 0.94 |
| 5th-95th percentile | 0.76 - 1.84 | 0.8 - 1.52 | 0.6 - 1.34 |
| %>LOD | 100 | 100 | 100 |
| **PC ae C40:2** |  |  |  |
| Median | 1.4 | 1.17 | 1.05 |
| 5th-95th percentile | 0.9 - 1.96 | 0.87 - 1.62 | 0.8 - 1.49 |
| %>LOD | 100 | 100 | 100 |
| **PC ae C40:3** |  |  |  |
| Median | 0.78 | 0.74 | 0.67 |
| 5th-95th percentile | 0.58 - 1.06 | 0.47 - 0.87 | 0.53 - 0.91 |
| %>LOD | 100 | 100 | 100 |
| **PC ae C40:4** |  |  |  |
| Median | 2.04 | 1.69 | 1.58 |
| 5th-95th percentile | 1.3 - 2.55 | 1.13 - 2.25 | 1.24 - 2.23 |
| %>LOD | 100 | 100 | 100 |
| **PC ae C40:5** |  |  |  |
| Median | 2.88 | 2.42 | 2.2 |
| 5th-95th percentile | 1.69 - 3.44 | 1.7 - 3.2 | 1.66 - 3.33 |
| %>LOD | 100 | 100 | 100 |
| **PC ae C40:6** |  |  |  |
| Median | 4.58 | 3.58 | 3.18 |
| 5th-95th percentile | 2.59 - 6.68 | 2.4 - 4.88 | 2.3 - 5.71 |
| %>LOD | 100 | 100 | 100 |
| **PC ae C42:0** |  |  |  |
| Median | 0.671 | 0.671 | 0.671 |
| 5th-95th percentile | 0.671 - 0.759 | 0.671 - 0.671 | 0.671 - 0.671 |
| %>LOD | 12 | 5 | 2 |
| **PC ae C42:1** |  |  |  |
| Median | 0.302 | 0.291 | 0.274 |
| 5th-95th percentile | 0.204 - 0.395 | 0.214 - 0.39 | 0.201 - 0.381 |
| %>LOD | 100 | 100 | 100 |
| **PC ae C42:2** |  |  |  |
| Median | 0.433 | 0.366 | 0.341 |
| 5th-95th percentile | 0.27 - 0.651 | 0.304 - 0.535 | 0.251 - 0.498 |
| %>LOD | 100 | 100 | 100 |
| **PC ae C42:3** |  |  |  |
| Median | 0.605 | 0.53 | 0.484 |
| 5th-95th percentile | 0.438 - 0.78 | 0.417 - 0.817 | 0.349 - 0.71 |
| %>LOD | 100 | 100 | 100 |
| **PC ae C42:4** |  |  |  |
| Median | 0.758 | 0.657 | 0.632 |
| 5th-95th percentile | 0.511 - 1.015 | 0.43 - 0.839 | 0.467 - 0.921 |
| %>LOD | 100 | 100 | 100 |
| **PC ae C42:5** |  |  |  |
| Median | 1.92 | 1.56 | 1.56 |
| 5th-95th percentile | 1.24 - 2.25 | 1.14 - 2.37 | 1.14 - 2.31 |
| %>LOD | 100 | 100 | 100 |
|  |  |  |  |
| **Metabolite^*^** | **Never**  **night shift workers** | **Former**  **night shift workers** | **Current**  **night shift workers** |
| **PC ae C44:3** |  |  |  |
| Median | 0.058 | 0.052 | 0.05 |
| 5th-95th percentile | 0.043 - 0.085 | 0.037 - 0.074 | 0.039 - 0.064 |
| %>LOD | 0 | 0 | 0 |
| **PC ae C44:4** |  |  |  |
| Median | 0.247 | 0.232 | 0.226 |
| 5th-95th percentile | 0.186 - 0.33 | 0.17 - 0.351 | 0.158 - 0.318 |
| %>LOD | 100 | 100 | 100 |
| **PC ae C44:5** |  |  |  |
| Median | 1.665 | 1.42 | 1.34 |
| 5th-95th percentile | 1.082 - 2.098 | 0.896 - 1.93 | 0.939 - 2.055 |
| %>LOD | 100 | 100 | 100 |
| **PC ae C44:6** |  |  |  |
| Median | 1.15 | 0.976 | 0.906 |
| 5th-95th percentile | 0.774 - 1.487 | 0.634 - 1.408 | 0.624 - 1.641 |
| %>LOD | 100 | 100 | 100 |
| **SM (OH) C14:1** |  |  |  |
| Median | 6.53 | 5.77 | 5.48 |
| 5th-95th percentile | 4.88 - 10.01 | 4.38 - 7.03 | 3.13 - 7.41 |
| %>LOD | 100 | 100 | 100 |
| **SM (OH) C16:1** |  |  |  |
| Median | 3.67 | 3.27 | 2.73 |
| 5th-95th percentile | 2.54 - 4.46 | 2.08 - 4.09 | 1.88 - 3.85 |
| %>LOD | 100 | 100 | 100 |
| **SM (OH) C22:1** |  |  |  |
| Median | 13.35 | 12.35 | 11.4 |
| 5th-95th percentile | 10.85 - 19.38 | 8.05 - 16.5 | 8.85 - 15.48 |
| %>LOD | 100 | 100 | 100 |
| **SM (OH) C22:2** |  |  |  |
| Median | 14.35 | 13.55 | 11.8 |
| 5th-95th percentile | 10.98 - 19.88 | 7.92 - 17.2 | 8.4 - 15.75 |
| %>LOD | 100 | 100 | 100 |
| **SM (OH) C24:1** |  |  |  |
| Median | 1.185 | 1.035 | 0.864 |
| 5th-95th percentile | 0.749 - 1.575 | 0.59 - 1.286 | 0.62 - 1.287 |
| %>LOD | 100 | 100 | 100 |
| **SM C16:0** |  |  |  |
| Median | 114 | 106.5 | 99 |
| 5th-95th percentile | 91 - 145.5 | 76.5 - 139.2 | 86.4 - 123.9 |
| %>LOD | 100 | 100 | 100 |
| **SM C16:1** |  |  |  |
| Median | 16.9 | 15.3 | 15 |
| 5th-95th percentile | 12.6 - 21.8 | 12.1 - 18.6 | 11.2 - 18.6 |
| %>LOD | 100 | 100 | 100 |
| **SM C18:0** |  |  |  |
| Median | 26.2 | 22.6 | 20.9 |
| 5th-95th percentile | 17.7 - 34.8 | 15.5 - 27.7 | 16.2 - 25.8 |
| %>LOD | 100 | 100 | 100 |
| **SM C18:1** |  |  |  |
| Median | 12.95 | 11.05 | 10.9 |
| 5th-95th percentile | 8.62 - 17.15 | 8.29 - 13.89 | 7.76 - 13.76 |
| %>LOD | 100 | 100 | 100 |
|  |  |  |  |
| **Metabolite^*^** | **Never**  **night shift workers** | **Former**  **night shift workers** | **Current**  **night shift workers** |
| **SM C20:2** |  |  |  |
| Median | 0.396 | 0.319 | 0.313 |
| 5th-95th percentile | 0.285 - 0.565 | 0.245 - 0.426 | 0.227 - 0.43 |
| %>LOD | 100 | 100 | 100 |
| **SM C22:3** |  |  |  |
| Median | 0.191 | 0.167 | 0.167 |
| 5th-95th percentile | 0.088 - 0.373 | 0.103 - 0.236 | 0.101 - 0.29 |
| %>LOD | 100 | 100 | 100 |
| **SM C24:0** |  |  |  |
| Median | 19.4 | 19.1 | 17.4 |
| 5th-95th percentile | 15.4 - 25.7 | 15.1 - 24.5 | 13.5 - 23.7 |
| %>LOD | 100 | 100 | 100 |
| **SM C24:1** |  |  |  |
| Median | 58.2 | 52.2 | 50.2 |
| 5th-95th percentile | 44.9 - 82.1 | 32.8 - 71.9 | 38.9 - 73.7 |
| %>LOD | 100 | 100 | 100 |
| **SM C26:0** |  |  |  |
| Median | 0.113 | 0.094 | 0.094 |
| 5th-95th percentile | 0.076 - 0.189 | 0.054 - 0.142 | 0.055 - 0.142 |
| %>LOD | 100 | 100 | 95 |
| **SM C26:1** |  |  |  |
| Median | 0.205 | 0.186 | 0.176 |
| 5th-95th percentile | 0.122 - 0.289 | 0.13 - 0.25 | 0.115 - 0.245 |
| %>LOD | 100 | 100 | 100 |
| **H1** |  |  |  |
| Median | 4713 | 4767 | 4484 |
| 5th-95th percentile | 3639 - 6108 | 3664 - 5885 | 3450 - 6124 |
| %>LOD | 100 | 95 | 100 |

**^*^Abbreviations:**

C0: L-Carnitine (Group: Acylcarnitines; Category: Acylcarnitines)

C10: Decanoylcarnitine (Group: Acylcarnitines; Category: Acylcarnitines)

C10:1: Decenoylcarnitine (Group: Acylcarnitines; Category: Acylcarnitines)

C10:2: Decadienylcarnitine (Group: Acylcarnitines; Category: Acylcarnitines)

C12: Dodecanoylcarnitine (Group: Acylcarnitines; Category: Acylcarnitines)

C12:1: Dodecenoylcarnitine (Group: Acylcarnitines; Category: Acylcarnitines)

C12-DC: Dodecanedioylcarnitine (Group: Acylcarnitines; Category: Acylcarnitines)

C14: Tetradecanoylcarnitine (Group: Acylcarnitines; Category: Acylcarnitines)

C14:1: Tetradecenoylcarnitine (Group: Acylcarnitines; Category: Acylcarnitines)

C14:1-OH: Hydroxytetradecenoylcarnitine (Group: Acylcarnitines; Category: Acylcarnitines)

C14:2: Tetradecadienylcarnitine (Group: Acylcarnitines; Category: Acylcarnitines)

C14:2-OH: Hydroxytetradecadienylcarnitine (Group: Acylcarnitines; Category: Acylcarnitines)

C16: Hexadecanoylcarnitine (Group: Acylcarnitines; Category: Acylcarnitines)

C16:1: Hexadecenoylcarnitine (Group: Acylcarnitines; Category: Acylcarnitines)

C16:1-OH: Hydroxyhexadecenoylcarnitine (Group: Acylcarnitines; Category: Acylcarnitines)

C16:2: Hexadecadienylcarnitine (Group: Acylcarnitines; Category: Acylcarnitines)

C16:2-OH: Hydroxyhexadecadienylcarnitine (Group: Acylcarnitines; Category: Acylcarnitines)

C16-OH: Hydroxyhexadecanoylcarnitine (Group: Acylcarnitines; Category: Acylcarnitines)

C18: Octadecanoylcarnitine (Group: Acylcarnitines; Category: Acylcarnitines)

C18:1: Octadecenoylcarnitine (Group: Acylcarnitines; Category: Acylcarnitines)

C18:1-OH: Hydroxyoctadecenoylcarnitine (Group: Acylcarnitines; Category: Acylcarnitines)

C18:2: Octadecadienylcarnitine (Group: Acylcarnitines; Category: Acylcarnitines)

C2: Acetylcarnitine (Group: Acylcarnitines; Category: Acylcarnitines)

C3: Propionylcarnitine (Group: Acylcarnitines; Category: Acylcarnitines)

C3:1: Propenoylcarnitine (Group: Acylcarnitines; Category: Acylcarnitines)

C3-DC (C4-OH): Hydroxybutyrylcarnitine (Group: Acylcarnitines; Category: Acylcarnitines)

C3-OH: Hydroxypropionylcarnitine (Group: Acylcarnitines; Category: Acylcarnitines)

C4: Butyrylcarnitine (Group: Acylcarnitines; Category: Acylcarnitines)

C4:1: Butenylcarnitine (Group: Acylcarnitines; Category: Acylcarnitines)

C5: Valerylcarnitine (Group: Acylcarnitines; Category: Acylcarnitines)

C5:1: Tiglylcarnitine (Group: Acylcarnitines; Category: Acylcarnitines)

C5:1-DC: Glutaconylcarnitine (Group: Acylcarnitines; Category: Acylcarnitines)

C5-DC (C6-OH): Glutarylcarnitine (Group: Acylcarnitines; Category: Acylcarnitines)

C5-M-DC: Methylglutarylcarnitine (Group: Acylcarnitines; Category: Acylcarnitines)

C5-OH (C3-DC-M): Hydroxyvalerylcarnitine (Group: Acylcarnitines; Category: Acylcarnitines)

C6 (C4:1-DC): Hexanoylcarnitine (Group: Acylcarnitines; Category: Acylcarnitines)

C6:1: Hexenoylcarnitine (Group: Acylcarnitines; Category: Acylcarnitines)

C7-DC: Pimelylcarnitine (Group: Acylcarnitines; Category: Acylcarnitines)

C8: Octanoylcarnitine (Group: Acylcarnitines; Category: Acylcarnitines)

C9: Nonaylcarnitine (Group: Acylcarnitines; Category: Acylcarnitines)

Ala: Alanine (Group: Aminoacids; Category: Aminoacids)

Arg: Arginine (Group: Aminoacids; Category: Aminoacids)

Asn: Asparagine (Group: Aminoacids; Category: Aminoacids)

Asp: Aspartic acid (Group: Aminoacids; Category: Aminoacids)

Cit: Citrulline (Group: Aminoacids; Category: Aminoacids)

Gln: Glutamine (Group: Aminoacids; Category: Aminoacids)

Glu: Glutamic acid (Group: Aminoacids; Category: Aminoacids)

Gly: Glycine (Group: Aminoacids; Category: Aminoacids)

His: Histidine (Group: Aminoacids; Category: Aminoacids)

Ile: Isoleucine (Group: Aminoacids; Category: Aminoacids)

Leu: Leucine (Group: Aminoacids; Category: Aminoacids)

Lys: Lysine (Group: Aminoacids; Category: Aminoacids)

Met: Methionine (Group: Aminoacids; Category: Aminoacids)

Orn: Ornithine (Group: Aminoacids; Category: Aminoacids)

Phe: Phenylalanine (Group: Aminoacids; Category: Aminoacids)

Pro: Proline (Group: Aminoacids; Category: Aminoacids)

Ser: Serine (Group: Aminoacids; Category: Aminoacids)

Thr: Threonine (Group: Aminoacids; Category: Aminoacids)

Trp: Tryptophan (Group: Aminoacids; Category: Aminoacids)

Tyr: Tyrosine (Group: Aminoacids; Category: Aminoacids)

Val: Valine (Group: Aminoacids; Category: Aminoacids)

Ac-Orn: N-Acetylornithine (Group: Biogenic Amines; Category: Biogenic Amines)

ADMA: Asymmetric dimethylarginine (Group: Biogenic Amines; Category: Biogenic Amines)

alpha-AAA: Aminoadipic acid (Group: Biogenic Amines; Category: Biogenic Amines)

c4-OH-Pro: cis-4-Hydroxyproline (Group: Biogenic Amines; Category: Biogenic Amines)

Carnosine: Carnosine (Group: Biogenic Amines; Category: Biogenic Amines)

Creatinine: Creatinine (Group: Biogenic Amines; Category: Biogenic Amines)

DOPA: DOPA (Group: Biogenic Amines; Category: Biogenic Amines)

Dopamine: Dopamine (Group: Biogenic Amines; Category: Biogenic Amines)

Histamine: Histamine (Group: Biogenic Amines; Category: Biogenic Amines)

Kynurenine: Kynurenine (Group: Biogenic Amines; Category: Biogenic Amines)

Met-SO: Methionine sulfoxide (Group: Biogenic Amines; Category: Biogenic Amines)

Nitro-Tyr: Nitrotyrosine (Group: Biogenic Amines; Category: Biogenic Amines)

PEA: Phenylethylamine (Group: Biogenic Amines; Category: Biogenic Amines)

Putrescine: Putrescine (Group: Biogenic Amines; Category: Biogenic Amines)

SDMA: Symmetric dimethylarginine (Group: Biogenic Amines; Category: Biogenic Amines)

Serotonin: Serotonin (Group: Biogenic Amines; Category: Biogenic Amines)

Spermidine: Spermidine (Group: Biogenic Amines; Category: Biogenic Amines)

Spermine: Spermine (Group: Biogenic Amines; Category: Biogenic Amines)

t4-OH-Pro: Hydroxyproline (Group: Biogenic Amines; Category: Biogenic Amines)

Taurine: Taurine (Group: Biogenic Amines; Category: Biogenic Amines)

total DMA: Total dimethylarginine (Group: Biogenic Amines; Category: Biogenic Amines)

lysoPC a C14:0: Lysophosphatidylcholine acyl C14:0 (Group: Glycerophospholipids; Category: Lysophosphatidylcholines)

lysoPC a C16:0: Lysophosphatidylcholine acyl C16:0 (Group: Glycerophospholipids; Category: Lysophosphatidylcholines)

lysoPC a C16:1: Lysophosphatidylcholine acyl C16:1 (Group: Glycerophospholipids; Category: Lysophosphatidylcholines)

lysoPC a C17:0: Lysophosphatidylcholine acyl C17:0 (Group: Glycerophospholipids; Category: Lysophosphatidylcholines)

lysoPC a C18:0: Lysophosphatidylcholine acyl C18:0 (Group: Glycerophospholipids; Category: Lysophosphatidylcholines)

lysoPC a C18:1: Lysophosphatidylcholine acyl C18:1 (Group: Glycerophospholipids; Category: Lysophosphatidylcholines)

lysoPC a C18:2: Lysophosphatidylcholine acyl C18:2 (Group: Glycerophospholipids; Category: Lysophosphatidylcholines)

lysoPC a C20:3: Lysophosphatidylcholine acyl C20:3 (Group: Glycerophospholipids; Category: Lysophosphatidylcholines)

lysoPC a C20:4: Lysophosphatidylcholine acyl C20:4 (Group: Glycerophospholipids; Category: Lysophosphatidylcholines)

lysoPC a C24:0: Lysophosphatidylcholine acyl C24:0 (Group: Glycerophospholipids; Category: Lysophosphatidylcholines)

lysoPC a C26:0: Lysophosphatidylcholine acyl C26:0 (Group: Glycerophospholipids; Category: Lysophosphatidylcholines)

lysoPC a C26:1: Lysophosphatidylcholine acyl C26:1 (Group: Glycerophospholipids; Category: Lysophosphatidylcholines)

lysoPC a C28:0: Lysophosphatidylcholine acyl C28:0 (Group: Glycerophospholipids; Category: Lysophosphatidylcholines)

lysoPC a C28:1: Lysophosphatidylcholine acyl C28:1 (Group: Glycerophospholipids; Category: Lysophosphatidylcholines)

PC aa C24:0: Diacylphosphatidylcholine C24:0 (Group: Glycerophospholipids; Category: Diacylphosphatidylcholines)

PC aa C26:0: Diacylphosphatidylcholine C26:0 (Group: Glycerophospholipids; Category: Diacylphosphatidylcholines)

PC aa C28:1: Diacylphosphatidylcholine C28:1 (Group: Glycerophospholipids; Category: Diacylphosphatidylcholines)

PC aa C30:0: Diacylphosphatidylcholine C30:0 (Group: Glycerophospholipids; Category: Diacylphosphatidylcholines)

PC aa C30:2: Diacylphosphatidylcholine C30:2 (Group: Glycerophospholipids; Category: Diacylphosphatidylcholines)

PC aa C32:0: Diacylphosphatidylcholine C32:0 (Group: Glycerophospholipids; Category: Diacylphosphatidylcholines)

PC aa C32:1: Diacylphosphatidylcholine C32:1 (Group: Glycerophospholipids; Category: Diacylphosphatidylcholines)

PC aa C32:2: Diacylphosphatidylcholine C32:2 (Group: Glycerophospholipids; Category: Diacylphosphatidylcholines)

PC aa C32:3: Diacylphosphatidylcholine C32:3 (Group: Glycerophospholipids; Category: Diacylphosphatidylcholines)

PC aa C34:1: Diacylphosphatidylcholine C34:1 (Group: Glycerophospholipids; Category: Diacylphosphatidylcholines)

PC aa C34:2: Diacylphosphatidylcholine C34:2 (Group: Glycerophospholipids; Category: Diacylphosphatidylcholines)

PC aa C34:3: Diacylphosphatidylcholine C34:3 (Group: Glycerophospholipids; Category: Diacylphosphatidylcholines)

PC aa C34:4: Diacylphosphatidylcholine C34:4 (Group: Glycerophospholipids; Category: Diacylphosphatidylcholines)

PC aa C36:0: Diacylphosphatidylcholine C36:0 (Group: Glycerophospholipids; Category: Diacylphosphatidylcholines)

PC aa C36:1: Diacylphosphatidylcholine C36:1 (Group: Glycerophospholipids; Category: Diacylphosphatidylcholines)

PC aa C36:2: Diacylphosphatidylcholine C36:2 (Group: Glycerophospholipids; Category: Diacylphosphatidylcholines)

PC aa C36:3: Diacylphosphatidylcholine C36:3 (Group: Glycerophospholipids; Category: Diacylphosphatidylcholines)

PC aa C36:4: Diacylphosphatidylcholine C36:4 (Group: Glycerophospholipids; Category: Diacylphosphatidylcholines)

PC aa C36:5: Diacylphosphatidylcholine C36:5 (Group: Glycerophospholipids; Category: Diacylphosphatidylcholines)

PC aa C36:6: Diacylphosphatidylcholine C36:6 (Group: Glycerophospholipids; Category: Diacylphosphatidylcholines)

PC aa C38:0: Diacylphosphatidylcholine C38:0 (Group: Glycerophospholipids; Category: Diacylphosphatidylcholines)

PC aa C38:1: Diacylphosphatidylcholine C38:1 (Group: Glycerophospholipids; Category: Diacylphosphatidylcholines)

PC aa C38:3: Diacylphosphatidylcholine C38:3 (Group: Glycerophospholipids; Category: Diacylphosphatidylcholines)

PC aa C38:4: Diacylphosphatidylcholine C38:4 (Group: Glycerophospholipids; Category: Diacylphosphatidylcholines)

PC aa C38:5: Diacylphosphatidylcholine C38:5 (Group: Glycerophospholipids; Category: Diacylphosphatidylcholines)

PC aa C38:6: Diacylphosphatidylcholine C38:6 (Group: Glycerophospholipids; Category: Diacylphosphatidylcholines)

PC aa C40:1: Diacylphosphatidylcholine C40:1 (Group: Glycerophospholipids; Category: Diacylphosphatidylcholines)

PC aa C40:2: Diacylphosphatidylcholine C40:2 (Group: Glycerophospholipids; Category: Diacylphosphatidylcholines)

PC aa C40:3: Diacylphosphatidylcholine C40:3 (Group: Glycerophospholipids; Category: Diacylphosphatidylcholines)

PC aa C40:4: Diacylphosphatidylcholine C40:4 (Group: Glycerophospholipids; Category: Diacylphosphatidylcholines)

PC aa C40:5: Diacylphosphatidylcholine C40:5 (Group: Glycerophospholipids; Category: Diacylphosphatidylcholines)

PC aa C40:6: Diacylphosphatidylcholine C40:6 (Group: Glycerophospholipids; Category: Diacylphosphatidylcholines)

PC aa C42:0: Diacylphosphatidylcholine C42:0 (Group: Glycerophospholipids; Category: Diacylphosphatidylcholines)

PC aa C42:1: Diacylphosphatidylcholine C42:1 (Group: Glycerophospholipids; Category: Diacylphosphatidylcholines)

PC aa C42:2: Diacylphosphatidylcholine C42:2 (Group: Glycerophospholipids; Category: Diacylphosphatidylcholines)

PC aa C42:4: Diacylphosphatidylcholine C42:4 (Group: Glycerophospholipids; Category: Diacylphosphatidylcholines)

PC aa C42:5: Diacylphosphatidylcholine C42:5 (Group: Glycerophospholipids; Category: Diacylphosphatidylcholines)

PC aa C42:6: Diacylphosphatidylcholine C42:6 (Group: Glycerophospholipids; Category: Diacylphosphatidylcholines)

PC ae C30:0: Acylalkylphosphatidylcholine C30:0 (Group: Glycerophospholipids; Category: Acylalkylphosphatidylcholines)

PC ae C30:1: Acylalkylphosphatidylcholine C30:1 (Group: Glycerophospholipids; Category: Acylalkylphosphatidylcholines)

PC ae C30:2: Acylalkylphosphatidylcholine C30:2 (Group: Glycerophospholipids; Category: Acylalkylphosphatidylcholines)

PC ae C32:1: Acylalkylphosphatidylcholine C32:1 (Group: Glycerophospholipids; Category: Acylalkylphosphatidylcholines)

PC ae C32:2: Acylalkylphosphatidylcholine C32:2 (Group: Glycerophospholipids; Category: Acylalkylphosphatidylcholines)

PC ae C34:0: Acylalkylphosphatidylcholine C34:0 (Group: Glycerophospholipids; Category: Acylalkylphosphatidylcholines)

PC ae C34:1: Acylalkylphosphatidylcholine C34:1 (Group: Glycerophospholipids; Category: Acylalkylphosphatidylcholines)

PC ae C34:2: Acylalkylphosphatidylcholine C34:2 (Group: Glycerophospholipids; Category: Acylalkylphosphatidylcholines)

PC ae C34:3: Acylalkylphosphatidylcholine C34:3 (Group: Glycerophospholipids; Category: Acylalkylphosphatidylcholines)

PC ae C36:0: Acylalkylphosphatidylcholine C36:0 (Group: Glycerophospholipids; Category: Acylalkylphosphatidylcholines)

PC ae C36:1: Acylalkylphosphatidylcholine C36:1 (Group: Glycerophospholipids; Category: Acylalkylphosphatidylcholines)

PC ae C36:2: Acylalkylphosphatidylcholine C36:2 (Group: Glycerophospholipids; Category: Acylalkylphosphatidylcholines)

PC ae C36:3: Acylalkylphosphatidylcholine C36:3 (Group: Glycerophospholipids; Category: Acylalkylphosphatidylcholines)

PC ae C36:4: Acylalkylphosphatidylcholine C36:4 (Group: Glycerophospholipids; Category: Acylalkylphosphatidylcholines)

PC ae C36:5: Acylalkylphosphatidylcholine C36:5 (Group: Glycerophospholipids; Category: Acylalkylphosphatidylcholines)

PC ae C38:0: Acylalkylphosphatidylcholine C38:0 (Group: Glycerophospholipids; Category: Acylalkylphosphatidylcholines)

PC ae C38:1: Acylalkylphosphatidylcholine C38:1 (Group: Glycerophospholipids; Category: Acylalkylphosphatidylcholines)

PC ae C38:2: Acylalkylphosphatidylcholine C38:2 (Group: Glycerophospholipids; Category: Acylalkylphosphatidylcholines)

PC ae C38:3: Acylalkylphosphatidylcholine C38:3 (Group: Glycerophospholipids; Category: Acylalkylphosphatidylcholines)

PC ae C38:4: Acylalkylphosphatidylcholine C38:4 (Group: Glycerophospholipids; Category: Acylalkylphosphatidylcholines)

PC ae C38:5: Acylalkylphosphatidylcholine C38:5 (Group: Glycerophospholipids; Category: Acylalkylphosphatidylcholines)

PC ae C38:6: Acylalkylphosphatidylcholine C38:6 (Group: Glycerophospholipids; Category: Acylalkylphosphatidylcholines)

PC ae C40:1: Acylalkylphosphatidylcholine C40:1 (Group: Glycerophospholipids; Category: Acylalkylphosphatidylcholines)

PC ae C40:2: Acylalkylphosphatidylcholine C40:2 (Group: Glycerophospholipids; Category: Acylalkylphosphatidylcholines)

PC ae C40:3: Acylalkylphosphatidylcholine C40:3 (Group: Glycerophospholipids; Category: Acylalkylphosphatidylcholines)

PC ae C40:4: Acylalkylphosphatidylcholine C40:4 (Group: Glycerophospholipids; Category: Acylalkylphosphatidylcholines)

PC ae C40:5: Acylalkylphosphatidylcholine C40:5 (Group: Glycerophospholipids; Category: Acylalkylphosphatidylcholines)

PC ae C40:6: Acylalkylphosphatidylcholine C40:6 (Group: Glycerophospholipids; Category: Acylalkylphosphatidylcholines)

PC ae C42:0: Acylalkylphosphatidylcholine C42:0 (Group: Glycerophospholipids; Category: Acylalkylphosphatidylcholines)

PC ae C42:1: Acylalkylphosphatidylcholine C42:1 (Group: Glycerophospholipids; Category: Acylalkylphosphatidylcholines)

PC ae C42:2: Acylalkylphosphatidylcholine C42:2 (Group: Glycerophospholipids; Category: Acylalkylphosphatidylcholines)

PC ae C42:3: Acylalkylphosphatidylcholine C42:3 (Group: Glycerophospholipids; Category: Acylalkylphosphatidylcholines)

PC ae C42:4: Acylalkylphosphatidylcholine C42:4 (Group: Glycerophospholipids; Category: Acylalkylphosphatidylcholines)

PC ae C42:5: Acylalkylphosphatidylcholine C42:5 (Group: Glycerophospholipids; Category: Acylalkylphosphatidylcholines)

PC ae C44:3: Acylalkylphosphatidylcholine C44:3 (Group: Glycerophospholipids; Category: Acylalkylphosphatidylcholines)

PC ae C44:4: Acylalkylphosphatidylcholine C44:4 (Group: Glycerophospholipids; Category: Acylalkylphosphatidylcholines)

PC ae C44:5: Acylalkylphosphatidylcholine C44:5 (Group: Glycerophospholipids; Category: Acylalkylphosphatidylcholines)

PC ae C44:6: Acylalkylphosphatidylcholine C44:6 (Group: Glycerophospholipids; Category: Acylalkylphosphatidylcholines)

SM (OH) C14:1: Hydroxysphingomyeline C14:1 (Group: Sphingolipids; Category: Sphingomyelins)

SM (OH) C16:1: Hydroxysphingomyeline C16:1 (Group: Sphingolipids; Category: Sphingomyelins)

SM (OH) C22:1: Hydroxysphingomyeline C22:1 (Group: Sphingolipids; Category: Sphingomyelins)

SM (OH) C22:2: Hydroxysphingomyeline C22:2 (Group: Sphingolipids; Category: Sphingomyelins)

SM (OH) C24:1: Hydroxysphingomyeline C24:1 (Group: Sphingolipids; Category: Sphingomyelins)

SM C16:0: Sphingomyeline C16:0 (Group: Sphingolipids; Category: Sphingomyelins)

SM C16:1: Sphingomyeline C16:1 (Group: Sphingolipids; Category: Sphingomyelins)

SM C18:0: Sphingomyeline C18:0 (Group: Sphingolipids; Category: Sphingomyelins)

SM C18:1: Sphingomyeline C18:1 (Group: Sphingolipids; Category: Sphingomyelins)

SM C20:2: Sphingomyeline C20:2 (Group: Sphingolipids; Category: Sphingomyelins)

SM C22:3: Sphingomyeline C22:3 (Group: Sphingolipids; Category: Sphingomyelins)

SM C24:0: Sphingomyeline C24:0 (Group: Sphingolipids; Category: Sphingomyelins)

SM C24:1: Sphingomyeline C24:1 (Group: Sphingolipids; Category: Sphingomyelins)

SM C26:0: Sphingomyeline C26:0 (Group: Sphingolipids; Category: Sphingomyelins)

SM C26:1: Sphingomyeline C26:1 (Group: Sphingolipids; Category: Sphingomyelins)

H1: sum of hexoses (Group: Sugars; Category: Sugars)
