# Supplementary material for: Metabolomic profiles in night shift workers: A cross-sectional study on hospital female nurses
Source: Front Public Health. 2023 Feb 23;11:1082074. doi: 10.3389/fpubh.2023.1082074 (PMC9999616; doi:10.3389/fpubh.2023.1082074)
Supplement: Supplementary file 1 [file Data_Sheet_1.ZIP › Borroni_NSW_Metabolome_SupplMaterial/Borroni_NSW_Metabolome_Table S3.docx]

**Table S3**: True negatives, true positives, false negatives, false positives, sensitivity and specificity means after the application of 5-fold cross-validation algorithm

|  | **True Negatives** | **True Positives** | **False Negatives** | **False**  **Positives** | **Sensitivity** | **Specificity** |
| --- | --- | --- | --- | --- | --- | --- |
| **Current shifters** | 3.60 | 7.60 | 1.60 | 1.00 | 0.84 | 0.83 |
| **Former shifters** | 3.80 | 2.60 | 1.40 | 1.80 | 0.70 | 0.69 |
| **Ever shifters** | 2.20 | 11.80 | 3.00 | 1.20 | 0.80 | 0.65 |
